# A New Family of Capsule Polymerases Generates Teichoic Acid-Like Capsule Polymers in Gram-Negative Pathogens

**DOI:** 10.1128/mBio.00641-18

**Published:** 2018-05-29

**Authors:** Christa Litschko, Davide Oldrini, Insa Budde, Monika Berger, Jochen Meens, Rita Gerardy-Schahn, Francesco Berti, Mario Schubert, Timm Fiebig

**Affiliations:** a Institute of Clinical Biochemistry, Hannover Medical School, Hannover, Germany; b GSK, Siena, Italy; c Institute for Microbiology, University of Veterinary Medicine Hannover, Hannover, Germany; d Department of Biosciences, University of Salzburg, Salzburg, Austria; Aachen University; Max Planck Institute for Infection Biology

**Keywords:** TagF, capsular polysaccharide, capsule, enzymatic synthesis, nuclear magnetic resonance, polymerases, polymers, teichoic acids, vaccines, veterinary vaccine development, Actinobacillus pleuropneumoniae, Haemophilus influenzae

## Abstract

Group 2 capsule polymers represent crucial virulence factors of Gram-negative pathogenic bacteria. They are synthesized by enzymes called capsule polymerases. In this report, we describe a new family of polymerases that combine glycosyltransferase and hexose- and polyol-phosphate transferase activity to generate complex poly(oligosaccharide phosphate) and poly(glycosylpolyol phosphate) polymers, the latter of which display similarity to wall teichoic acid (WTA), a cell wall component of Gram-positive bacteria. Using modeling and multiple-sequence alignment, we showed homology between the predicted polymerase domains and WTA type I biosynthesis enzymes, creating a link between Gram-negative and Gram-positive cell wall biosynthesis processes. The polymerases of the new family are highly abundant and found in a variety of capsule-expressing pathogens such as Neisseria meningitidis, Actinobacillus pleuropneumoniae, Haemophilus influenzae, Bibersteinia trehalosi, and Escherichia coli with both human and animal hosts. Five representative candidates were purified, their activities were confirmed using nuclear magnetic resonance (NMR) spectroscopy, and their predicted folds were validated by site-directed mutagenesis.

## IMPORTANCE

Bacterial capsules play an important role in the interaction between a pathogen and the immune system of its host. During the last decade, capsule polymerases have become attractive tools for the production of capsule polymers applied as antigens in glycoconjugate vaccine formulations. Conventional production of glycoconjugate vaccines requires the cultivation of the pathogen and thus the highest biosafety standards, leading to tremendous costs. With regard to animal husbandry, where vaccines could avoid the extensive use of antibiotics, conventional production is not sufficiently cost-effective. In contrast, enzymatic synthesis of capsule polymers is pathogen-free and fast, offers high stereo- and regioselectivity, and works with high efficacy. The new capsule polymerase family described here vastly increases the toolbox of enzymes available for biotechnology purposes. Representatives are abundantly found in human pathogens but also in animal pathogens, paving the way for the exploitation of polymerases for the development of a new generation of vaccines for animal husbandry.

## INTRODUCTION

Bacterial pathogens have developed a variety of strategies to ensure their survival in a host (1). Among these strategies is the expression of a capsule consisting of extracellular polymers that form an extensive protective layer (1, 2). Capsules are widely distributed and found in diverse pathogens such as Escherichia coli, Neisseria meningitidis, Haemophilus influenzae, Actinobacillus pleuropneumoniae, Staphylococcus aureus, and Streptococcus pneumoniae (1, 3).

On the basis of the genetic and chemical properties of different E. coli strains, capsules are divided into four groups (4). Group 2 capsules consist of linear polymers displaying a high negative-charge density that is introduced by either negatively charged sugar residues (sialic acid or glucuronic acid) or phosphate groups (1).

**FIG 1:**
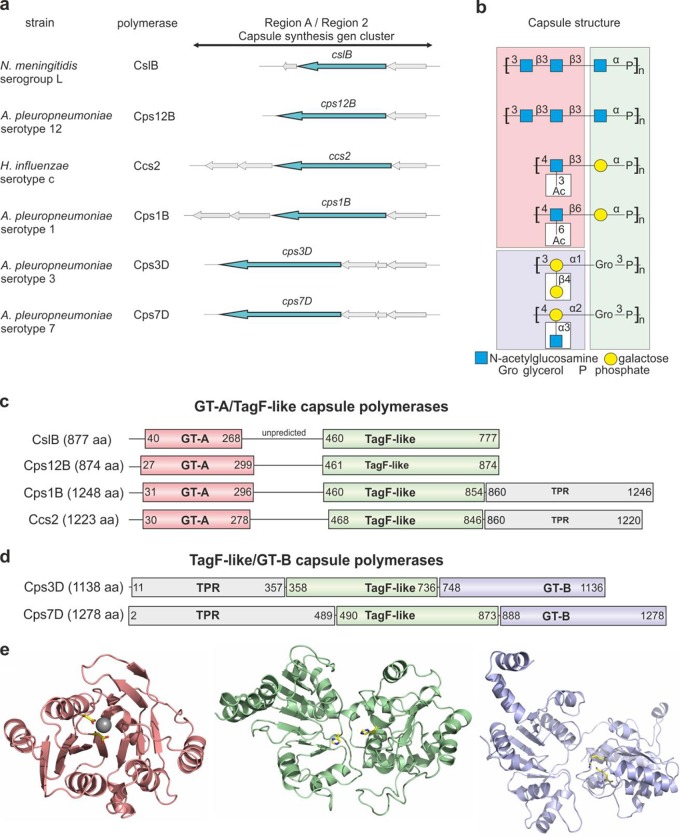
The TagF-like capsule polymerase family. (a) List of TagF-like polymerases characterized in this study and corresponding bacterial strains and schematic representation of regions A and 2 of their capsule gene cluster. Polymerase-encoding genes are highlighted in turquoise. (b) Polymer structures generated by TagF-like polymerases consist of linear backbones. Magenta, violet, and green background colors indicate the domains that are most likely to transfer the respective moiety based on the activity of the templates used for PHYRE2 modeling. Modifications (white boxes) are introduced by separate and as-yet-unidentified enzymes. (c and d) Schematic domain organization of (c) GT-A/TagF and (d) TagF/GT-B folded polymerases. The length of each polypeptide in amino acids (aa; indicated in parentheses after each name) as well as the sequence coverage of each modeled domain is indicated. The majority of polymerases contain a domain rich in tetratricopeptide repeats (TPR) (see Fig. 6). (e) From left to right: homology model for the GT-A domain (magenta) of Cps1B showing the aspartates of the DxD motif coordinating a Mg^2+^ ion; homology model for the TagF-like domain (green) of Cps7D depicting the two catalytically important histidine residues; homology model of the GT-B domain (violet) showing the conserved lysine and arginine residues. The importance of the highlighted active-site amino acids (yellow) is demonstrated in Fig. 7.

Genes required for biosynthesis and export of group 2 capsules are located in the so-called capsule gene cluster (1, 4–10). It is structured as three regions, of which regions 1 and 3 are conserved and encode proteins responsible for initiating capsule biosynthesis and transporting the polymers to the cell surface (see Fig. S1 in the supplemental material). Region 2 contains the serogroup-specific capsule polymerases (referred to here as “polymerases”) that assemble the respective capsule polymers (Fig. 1a). (Capsule polymers are often referred to as "capsule polysaccharides," especially when they consist exclusively of saccharide units.)

10.1128/mBio.00641-18.1 FIG S1  Schematic overview of capsule gene clusters. (a) E. coli K2 (E. L. Buckles, X. Wang, M. C. Lane, C. V. Lockatell, D. E. Johnson, D. A. Rasko, H. L. T. Mobley, and M. S. Donnenberg, J. Infect. Dis. **199:**1689–1697, 2009). (b) *N. meningitidis* serogroup L (O. B. Harrison, H. Claus, Y. Jiang, J. S. Bennett, H. B. Bratcher, K. A. Jolley, C. Corton, R. Care, J. T. Poolman, W. D. Zollinger, C. E. Frasch, D. S. Stephens, I. Feavers, M. Frosch, J. Parkhill, U. Vogel, M. A. Quail, S. D. Bentley, and M. C. Maiden, Emerg. Infect. Dis. **19:**566–573, 2013). (c) *A. pleuropneumoniae* serotypes 1, 3, 7, and 12 (H. Ito, J. Vet. Med. Sci. **77:**583–586, 2015; S. G. Jessing, P. Ahrens, T. J. Inzana, and Ø. Angen, Vet. Microbiol. **129:**350–359, 2008; Z. Xu, X. Chen, L. Li, T. Li, S. Wang, H. Chen, and R. Zhou, J. Bacteriol. **192:**5625–5636, 2010). (d) H. influenzae serotype c (S. W. Satola, P. L. Schirmer, and M. M. Farley, Infect. Immun. **71:**3639–3644, 2003; T.-T. Lâm, H. Claus, M. Frosch, and U. Vogel, Res. Microbiol. **162:**483–487, 2011; S. Sukupolvi-Petty, S. Grass, and J. W. St. Geme III, J. Bacteriol. **188:**3870–3877, 2006). The gene clusters are divided into conserved regions (gray boxes) and capsule-specific regions (white boxes). The conserved regions encode proteins necessary for translocation (green) and export (blue) of the capsule polymer to the cell surface. Genes encoding capsule polymerases are highlighted in red and localized in the capsule-specific region. Genes and interspaces in this scheme are not drawn to scale. The graphical representation follows the style used in a previous publication (B. F. Cress, J. A. Englaender, W. He, D. Kasper, R. J. Linhardt, and M. A. G. Koffas, FEMS Microbiol. Rev. **38:**660–697, 2014). Download FIG S1, PDF file, 0.3 MB. Copyright © 2018 Litschko et al. 2018 Litschko et al. This content is distributed under the terms of the Creative Commons Attribution 4.0 International license.

The polymerases described so far can be classified on the basis of their catalytic activity as glycosyltransferases (GTs) and hexose-1-phosphate transferases. Polymerases with GT activity generate glycosidic linkages in polymers consisting exclusively of saccharide units. On the structural level, the catalytic domains of these enzymes adopt one of the two most abundant GT folds, i.e., either GT-A or GT-B. The characteristic elements of both folds are two Rossmann-like domains that are either tightly associated, forming a central, continuous β-sheet (GT-A), or opposed to each other, forming a deep cleft that contains the catalytic center (GT-B) (11, 12). Polymerases with GT activity are either single-domain enzymes acting alone to synthesize a homopolymer (13–16) or, when generating a heteropolymer, acting in concert with another domain of the same GT fold type, which is either independently expressed (1, 17) or part of the same polypeptide (18–21).

Polymerases with hexose-1-phosphate transferase activity assemble a polymer in which monosaccharides are bridged by phosphodiester linkages (22–24). No structural and little mechanistic data (25–27) are available for these polymerases yet, but they are believed to be single-domain enzymes displaying sequence motifs that are characteristic of the members of a protein family called *stealth* (28).

With enzyme CslB of *N. meningitidis* serogroup L, we recently described for the first time a polymerase which is able to introduce both linkage types into one polymer, generating a structure consisting of trisaccharides connected by phosphodiester linkages (29). Despite its phosphotransferase activity, CslB shows no similarity to the hexose-1-phosphate transferases of the members of the *stealth* protein family. Instead, it exhibits a so far unique bipartite architecture consisting of an N-terminal GT-A fold and a C-terminal GT-B-like fold (29).

In the current study, we searched for homologues of CslB and identified a set of multidomain polymerases in a variety of animal and human pathogens. One subset of these polymerases was found to synthesize, like CslB, a poly(oligosaccharide phosphate) capsule. A second subset, however, was shown to assemble a poly(glycosylpolyol phosphate) polymer that displays considerable similarity to wall teichoic acid (WTA) type II, a negatively charged polymer and major constituent of the Gram-positive cell envelope. (Note that the terms "polyol" and "alditol" are often used synonymously in literature describing WTA.) In line with this finding, bioinformatics analysis of all identified primary protein sequences revealed as a common denominator a domain that showed sequence similarity to TagF, the wall teichoic acid synthase from Staphylococcus epidermidis and the best-characterized member of the so-called TagF-like protein family. Using site-directed mutagenesis, high-performance liquid chromatography (HPLC), and nuclear magnetic resonance (NMR) spectroscopy, we confirmed the modeling data and the hypothesized enzymatic activity.

## RESULTS

### Identification of the TagF-like polymerase family.

It is known that glycosyltransferases with similar activities can show low sequence identity (30, 31). Consequently, we searched for homologues of CslB not only by performing protein BLAST (32) searches using the CslB amino acid sequence (AEQ62070.1) but also by manually searching the literature for complex, phosphate-containing group 2 capsules with structures similar to that of the poly(oligosaccharide phosphate) polymer expressed by *N. meningitidis* serogroup L (Fig. 1b; see also Fig. S2 in the supplemental material). The best score (99% query cover, 58% sequence identity) in the protein BLAST search was obtained for putative glycosyltransferase Cps12B (AAS77491.1) from A. pleuropneumoniae serotype 12, a pathogen expressing a capsule identical to the polymer generated by CslB (29, 33). Also among the hits was Ccs2 (AEC50903.1; 97% query cover, 33% sequence identity) from H. influenzae serotype c, which expresses a capsule consisting of disaccharide repeating units connected through phosphodiester linkages (34). Literature searches revealed that similar dimeric units are expressed by *A. pleuropneumoniae* serotypes 1 (35) and 4 (36) and by H. influenzae serotype f (37, 38). Moreover, in a multitude of bacterial species, one of the hexoses of these dimeric repeating units is replaced by a glycerol, creating a structure similar to that of WTA type II (39, 40) (Fig. 1b; see also Fig. S2). Among those species are *N. meningitidis* serogroup H (41) and serogroup Z (42), E. coli K2 (43), and *A. pleuropneumoniae* serotypes 2 (44), 3 (45), 7 (46), 9 (47), and 11 (48) as well as Bibersteinia trehalosi (formerly *Mannheimia* [*Pasteurella*] *haemolytica* [49]) serotypes T3 (50), T4 (51), and T15 (52). Following the hypothesis that all of the linear polymer backbones mentioned above are generated by homologues of CslB (note that modifications of the linear backbone as shown in Fig. 1b [see also Fig. S2] are usually introduced by separate enzymes [1]), we analyzed the DNA sequence information from the corresponding strains and identified putative polymerases in the capsule gene cluster by their unusual length of more than 2,500 bp per open reading frame (ORF) (Fig. 1a; see also Fig. S1). Homology modeling, performed for each polypeptide sequence using the structure prediction software PHYRE2 (53), revealed that all putative polymerases, like CslB, contained as a common denominator a domain that was modeled with 100% confidence onto the crystal structure of TagF, the wall teichoic acid (type I) synthase of the Gram-positive bacterium Staphylococcus epidermidis. The corresponding domain is referred to here as TagF-like domain (see Fig. 1c to e; see also Fig. S3) (54). TagF itself is the most extensively studied member of the TagF-like protein family, which is characterized by five active-site primary sequence motifs (54, 55) and has so far been biochemically investigated using only Gram-positive sources. Despite their Gram-negative origin and the highly differing percentages of sequence identity with respect to their TagF-like domains (15% to 93%; see Fig. S4), all polymerases identified here contained the five active-site motifs as shown by multiple-sequence alignment using the Clustal Omega algorithm (56) (Fig. S5). It thus seems reasonable to allocate them to the TagF-like protein family and classify them as group 2 TagF-like polymerases.

10.1128/mBio.00641-18.2 FIG S2  Capsule structures of group 2 capsule-expressing bacteria that encode TagF-like polymerases. Branching mono- and oligosaccharides as well as O-acetyl groups are usually introduced by separate enzymes. Schematics of wall teichoic acid (WTA) types I and II are depicted for comparison and displayed in accordance with the style used in a previous publication (I. B. Naumova, A. S. Shashkov, E. M. Tul’skaya, G. M. Streshinskaya, Y. I. Kozlova, N. V. Potekhina, L. I. Evtushenko, and E. Stackebrandt, FEMS Microbiol. Rev. **25:**269–284, 2001). To allow a concise display, bacterial species are abbreviated in italics and serogroup/serotype classification data are added in regular font. The abbreviations used are as follows: *App*, Actinobacillus pleuropneumoniae; *Bt*, Bibersteinia trehalosi; *Hi*, Haemophilus influenzae; *Nm*, Neisseria meningitidis. Download FIG S2, PDF file, 0.2 MB. Copyright © 2018 Litschko et al. 2018 Litschko et al. This content is distributed under the terms of the Creative Commons Attribution 4.0 International license.

10.1128/mBio.00641-18.3 FIG S3  Overview of the predicted architecture of all TagF-like polymerases analyzed in this study. Homology modeling was performed using the structure prediction tool PHYRE2 (L. A. Kelley, S. Mezulis, C. M. Yates, M. N. Wass, and M. J. E. Sternberg, Nat. Protoc. **10:**845–858, 2015.). The name of the polymerase is displayed in front of each model. A ruler (bottom) indicates the length of each polypeptide as well as the sequence coverage of each modeled domain. Sequences of the following proteins were submitted to PHYRE2: CslB of *N. meningitidis* serogroup L (UniProt: Q9RGQ9), Cps1B of A. pleuropneumoniae serotype 1 (UniProt: E0EA77), Cps12B of A. pleuropneumoniae serotype 12 (UniProt: Q69AA8), Ccs2 of H. influenzae serotype c (GenBank accession number AEC50903.1), Fcs2 of H. influenzae serotype f (GenBank accession number AAQ12660.1), Cps4B of A. pleuropneumoniae serotype 4 (UniProt: F4YBG0), BtY31 of nonserotyped Bibersteinia trehalosi strain Y31 (GenBank accession number OAQ14264.1), Cps7D of A. pleuropneumoniae serotype 7 (GenBank accession number ACE62291.1), Cps2D of A. pleuropneumoniae serotype 2 (UniProt: Q6UYC4), CszC of *N. meningitidis* serogroup Z (UniProt: Q5QRV6), Cps3D of A. pleuropneumoniae serotype 3 (GenBank accession number KY807157), Cps9D of A. pleuropneumoniae serotype 9 (UniProt: E0F019), CshC of *N. meningitidis* serogroup H (UniProt: H6T5X6), Cps11D of A. pleuropneumoniae serotype 11 (UniProt: E0FCQ3), Bt188 of nonserotyped Bibersteinia trehalosi strain USDA-ARS-USMARC-188 (GenBank accession number AHG82487.1), Bt189 of nonserotyped Bibersteinia trehalosi strain USDA-ARS-USMARC-189 (GenBank accession number AHG84818.1), Bt192 of nonserotyped Bibersteinia trehalosi strain USDA-ARS-USMARC-192 (GenBank accession number AGH37704.1), and c3694 of E. coli K2 strain CFT073 (GenBank accession number AAN82142.1). Download FIG S3, PDF file, 0.2 MB. Copyright © 2018 Litschko et al. 2018 Litschko et al. This content is distributed under the terms of the Creative Commons Attribution 4.0 International license.

10.1128/mBio.00641-18.4 FIG S4  Sequence identity matrix (with data expressed in percentages) based on a Clustal Omega multiple-sequence alignment of all predicted TagF-like domains (as they are shown in Fig. S3) and the TagF modeling template. Download FIG S4, PDF file, 0.04 MB. Copyright © 2018 Litschko et al. 2018 Litschko et al. This content is distributed under the terms of the Creative Commons Attribution 4.0 International license.

10.1128/mBio.00641-18.5 FIG S5  Sequence alignment of all predicted TagF-like domains analyzed in this study, including the sequence of the TagF template of Staphylococcus epidermidis (UniProt: Q5HLM5) used for PHYRE2 modeling. Database references for all TagF-like polymerase sequences are indicated in the figure legend of Fig. S3. Identical amino acids are shown in gray boxes, and the conserved histidine residues are highlighted in red. The conserved active-site motifs reported for TagF are shown in boxed sections (red). The sequence alignment was performed with Clustal Omega (F. Sievers, A. Wilm, D. Dineen, T. J. Gibson, K. Karplus, W. Li, R. Lopez, H. McWilliam, M. Remmert, J. Söding, J. D. Thompson, and D. G. Higgins, Mol. Syst. Biol. **7:**539, 2011) on the UniProt website (http://www.uniprot.org/align/) (E. , D. Lieberherr, M. Tognolli, M. Schneider, and A. Bairoch, Methods Mol. Biol. **406:**89–112, 2007) and annotated with Jalview software (A. M. Waterhouse, J. B. Procter, D. M. A. Martin, M. Clamp, and G. J. Barton, Bioinformatics **25:**1189–1191, 2009). Download FIG S5, PDF file, 1.8 MB. Copyright © 2018 Litschko et al. 2018 Litschko et al. This content is distributed under the terms of the Creative Commons Attribution 4.0 International license.

In addition to the TagF-like domain, all models were predicted to have either a GT-A folded domain at the N terminus or a GT-B folded domain C-terminally flanking the TagF-like domain (Fig. 1c to e; see also Fig. S3). It is of note that the GT-A fold, modeled onto the β-glycosyltransferase domain of capsule polymerase K4CP from E. coli K4 (57), was present only in strains expressing a capsule with β-glycosidic linkage, whereas the GT-B fold, modeled onto the teichoic acid modifying α-glycosyltransferase TarM from Staphylococcus aureus (58), was found only in strains expressing a polymer with α-glycosidic linkage (Fig. 1b to e; domains and residues are highlighted in magenta and violet). Despite the considerably differing percentages of sequence identity (30% to 70% for GT-A and 40% to 90% for GT-B; see Fig. S6), all GT domains were modeled by PHYRE2 with 100% confidence. The two resulting archetypal polymerase architectures are referred to here as GT-A/TagF-like and TagF-like/GT-B architectures (see Fig. 1c and d).

10.1128/mBio.00641-18.6 FIG S6  Sequence identity matrices (with data expressed in percentages) based on a Clustal Omega multiple-sequence alignment of (a) all predicted GT-A domains (as they are shown in Fig. S3) and the C-terminal domain of the K4CP modeling template (E. coli K4 polymerase) and (b) all predicted GT-B domains (as they are shown in Fig. S3) and the TarM modeling template. Download FIG S6, PDF file, 0.1 MB. Copyright © 2018 Litschko et al. 2018 Litschko et al. This content is distributed under the terms of the Creative Commons Attribution 4.0 International license.

### Functional testing of recombinant capsule polymerases.

**FIG 2:**
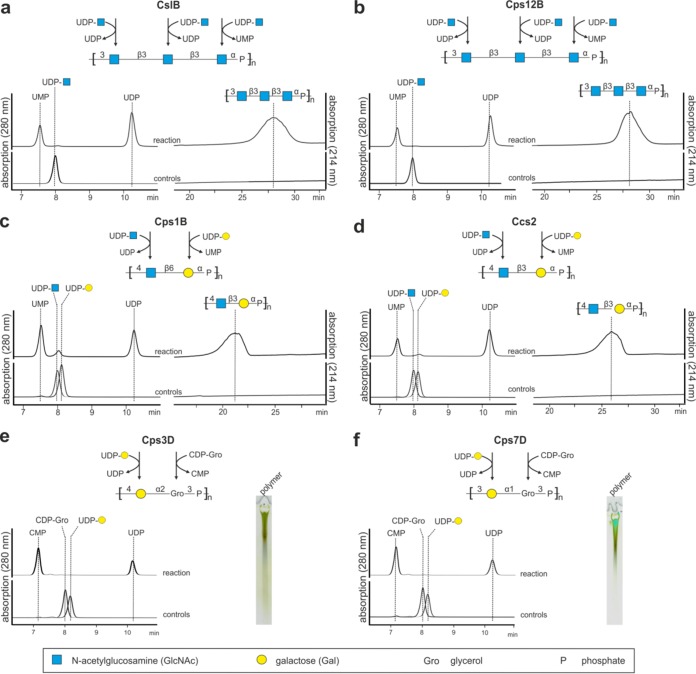
HPLC-AEC and PAGE analyses of different polymerase reactions. (a) CslB. (b) Cps12B. (c) Cps1B. (d) Ccs2. (e) Cps3D. (f) Cps7D. The HPLC-AEC assay allows the separation and detection of nucleotide activated donor substrates and released nucleotide products in the 280-nm channel (left panels). UV-active polymers carrying GlcNAc moieties were detected in the 214-nm channel (see panels a to d). UV-inactive polymers were detected using alcian blue/silver-stained PAGE (e and f).

To corroborate the predicted activity of the TagF-like polymerase family, five representative candidates were selected for further analyses. Cps12B was chosen due to its high similarity to CslB (29) (Fig. 1b and c). Cps1B and Ccs2 were additionally selected from the GT-A/TagF-like subgroup since they generate dimeric instead of trimeric repeating units (Fig. 1b and c), and Cps3D and Cps7D were selected from the TagF-like/GT-B subgroup as candidates synthesizing WTA-like products (Fig. 1b and d). The ORFs coding for the putative polymerases were amplified from bacterial lysates, cloned with different N- and C-terminal tags (maltose binding protein [MBP] and/or hexahistidine [His_6_], respectively), and expressed in E. coli. For each polymerase, the constructs yielding the best expression levels (namely, MBP-Cps12B-His_6_, MBP-Cps1B-His_6_, MBP-Cps3D-His_6_, MBP-Cps7D-His_6_, and Ccs2-His_6_; for concise presentation of the data, all constructs are displayed here without tags) were purified by affinity chromatography and subsequent size exclusion chromatography (SEC) (Fig. S7). Enzymatic activity was tested by incubating each enzyme with its putative donor substrates (nucleotide-activated monosaccharides or alditols, e.g., UDP-GlcNAc [UDP N-acetylglucosamine] or CDP-glycerol) in reaction buffer (29) supplemented with magnesium chloride. After 3 h, the reactions were analyzed using a high-performance liquid chromatography–anion exchange chromatography (HPLC-AEC) assay. In all reactions, the consumption of the donor substrates and the simultaneous formation of the nucleotide products (UMP, CMP, UDP) could be observed in the 280-nm channel (compare control and reaction data in Fig. 2). Simultaneously, the formation of polymer in Cps12B, Cps1B, and Ccs2 reactions was detected in the 214-nm channel, whereas polymers generated by Cps3D and Cps7D, lacking the UV-active N-acetyl group, were visualized by alcian blue/silver-stained polyacrylamide gel electrophoresis (PAGE) (Fig. 2). It is of note that all reactions took place in the absence of priming acceptor substrates (e.g., hydrolyzed polymer), demonstrating that all polymerases were able to initiate polymer synthesis *de novo*. To evaluate the relevance of Mg^2+^ for the activity of the TagF-like polymerases, the reactions described above were repeated in the presence of the Mg^2+^-chelating agent ethylenediaminetetraacetate (EDTA). In agreement with the findings indicating that GT-A folded proteins are dependent on divalent cations for the coordination of the negatively charged donor substrates (11) whereas basic amino acids assume this function in GT-B folded proteins (12), polymerases with the TagF-like/GT-B architecture were active even in the presence of EDTA, while the activity of polymerases adopting the GT-A/TagF-like architecture strictly depended on the presence of Mg^2+^ (Fig. S8).

10.1128/mBio.00641-18.7 FIG S7  Coomassie-stained SDS-polyacrylamide gel of TagF-like polymerases purified by affinity chromatography via their C-terminal His_6_ tag. N-terminal fusion to maltose binding protein is indicated with “MBP” in the construct name. A total of 1.5 to 3 μg of protein was loaded per lane. MBP-Cps3D-His_6_ (177 kDa) and MBP-Cps7D-His_6_ (192 kDa) could be enriched as full-length constructs. Western blot analysis performed with an anti-MBP antibody (data not shown) demonstrated that MBP-Cps1B-His_6_ (190 kDa), MBP-Cps12B-His_6_ (146 kDa), and Ccs2-His_6_ (144 kDa) were exclusively purified as N-terminal degradation products (indicated by arrows) lacking the MBP tag. N-terminal degradation is common for group 2 polymerases and usually does not interfere with activity (T. Fiebig, F. Freiberger, V. Pinto, M. R. Romano, A. Black, C. Litschko, A. Bethe, D. Yashunsky, R. Adamo, A. Nikolaev, F. Berti, and R. Gerardy-Schahn, J. Biol. Chem. **289:**19395–19407, 2014; C. Litschko, M. R. Romano, V. Pinto, H. Claus, U. Vogel, F. Berti, R. Gerardy-Schahn, and T. Fiebig, J. Biol. Chem. **290:**24355–24366, 2015). The dominant protein band purified from the MBP-Cps1B-His_6_ expression culture was N-terminally sequenced and could be identified as a Cps1B ΔN31 truncation. The corresponding construct Cps1B_32–1246_-His_6_ (144 kDa) was cloned and purified as well as the N- and C-terminally truncated construct Cps1B_32–858_-His_6_ (99 kDa) lacking the TPR domain. M, marker; His_6_, hexahistidine tag. Download FIG S7, PDF file, 0.1 MB. Copyright © 2018 Litschko et al. 2018 Litschko et al. This content is distributed under the terms of the Creative Commons Attribution 4.0 International license.

10.1128/mBio.00641-18.8 FIG S8  HPLC-AEC assay performed in the presence (+MgCl_2_) and absence (+EDTA) of magnesium chloride. (a) Polymerases containing GT-A folded domains depend on Mg^2+^ to stabilize the negative charge of the diphosphate of their donor substrate (C. Breton, L. Snajdrová, C. Jeanneau, J. Koca, and A. Imberty, Glycobiology **16:**29R–37R, 2006). (b) Consistent with the fact that there is no evidence of a bound metal ion associated with catalysis in GT-B folded enzymes (C. Breton, L. Snajdrová, C. Jeanneau, J. Koca, and A. Imberty, Glycobiology **16:**29R–37R, 2006), polymerases adopting the TagF-like/GT-B architecture also work in the presence of the chelating agent EDTA. Download FIG S8, PDF file, 0.3 MB. Copyright © 2018 Litschko et al. 2018 Litschko et al. This content is distributed under the terms of the Creative Commons Attribution 4.0 International license.

With the aim of analyzing the structure of the *in vitro*-synthesized polymers by NMR spectroscopy, the enzymatic reactions presented in Fig. 2 were upscaled and preparative AEC was used for polymer purification.

### NMR analysis confirms the predicted activity of Cps1B, Cps12B, and Ccs2.

**FIG 3:**
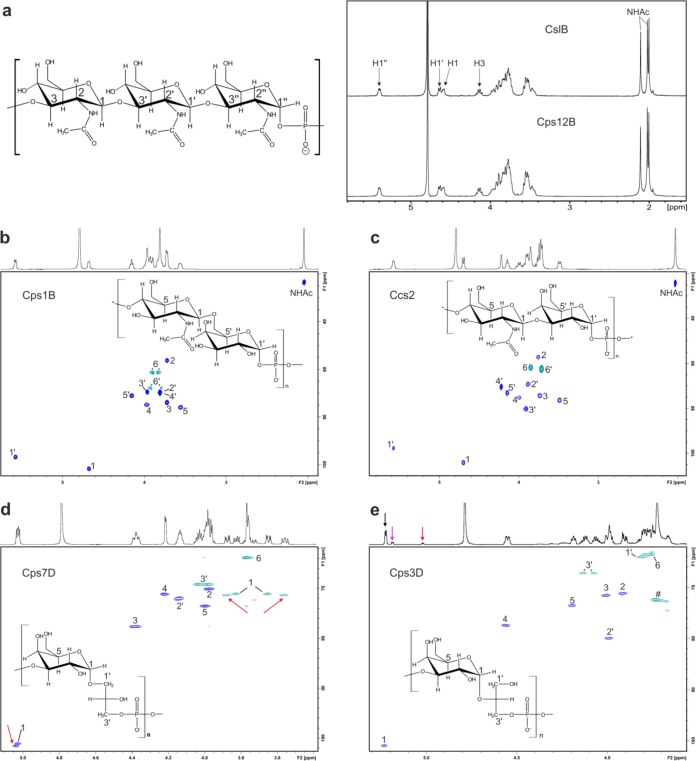
NMR characterization of the enzymatically synthesized polymers. (a) ^1^H NMR comparison of the Cps12B reaction product and the previously characterized polymer assembled by CslB. (b) ^1^H, ^13^C HSQC of the polymer generated by Cps1B. (c) ^1^H, ^13^C HSQC of the polymer generated by Ccs2. (d) ^1^H, ^13^C HSQC of the polymer generated by Cps7D. Red arrows indicate correlations that are not in agreement with the previously published spectra for the capsule polymer of A. pleuropneumoniae serotype 7. (e) ^1^H, ^13^C HSQC of the polymer generated by Cps3D. The signal-to-noise ratio was adjusted to show only the resonances belonging to the dominant spin system containing the anomeric signal with the highest (76%) intensity (black arrow), which is in agreement with the previously published structure of the A. pleuropneumoniae serotype 3 capsule polymer. The two additional anomeric signals (14% and 10%) resulting from the incorporation of the nonnatural substrate sn-glycerol-1-phosphate are indicated by red and magenta arrows. Signals labeled with a crosshatch symbol (#) are due to process-related impurities which were absent in a second batch of Cps3D product (data not shown).

The identity between the polymer generated by Cps12B and the previously characterized polymer generated by CslB (29) could be readily demonstrated by ^1^H NMR spectroscopy (Fig. 3a), confirming that Cps12B is the polymerase of A. pleuropneumoniae serotype 12. ^1^H, ^13^C heteronuclear single-quantum correlation (HSQC) experiments were performed to characterize the products generated by Cps1B and Ccs2 and demonstrated that the obtained ^13^C chemical shift values (Fig. 3b and c) (Table 1) were in perfect agreement with ^13^C spectra of the native, de-O-acetylated capsule polymer of A. pleuropneumoniae serotype 1 (35) and H. influenzae serotype c (34), respectively.

**TABLE 1:** ^13^C chemical shifts of the polymers generated by Cps1B, Ccs2, Cps7D, and Cps3D, derived from the ^1^H, ^13^C HSQC experiment represented in Fig. 3^a^

Product	^13^C chemical shifts (ppm)											
	Glucosamine						Galactose					
	C_1_	C_2_	C_3_	C_4_	C_5_	C_6_	C_1_′	C_2_′	C_3_′	C_4_′	C_5_′	C_6_′
Cps1B product	101.7	56.3	73.9	74.8	75.9	61.3	96.8	69.1	69.4	70.0	71.0	67.8
Ccs2 product	104.3	57.1	74.1	75.0	76.2	61.6	97.7	69.0	79.9	70.3	73.0	62.2
	Galactose						Glycerol					
	C_1_	C_2_	C_3_	C_4_	C_5_	C_6_	C_1_′	C_2_′	C_3_′			
Cps7D product	101.1	70.1	77.7	71.2	73.6	63.9	71.1	72.0	69.2			
Cps3D product	100.9	71.2	71.6	77.4	73.5	63.6	64.0	79.9	67.2			

a For the Cps7D and Cps3D products, only the shifts belonging to the naturally identical sn-glycerol-3-phosphate-containing repeating units are shown.

### NMR analysis confirms the predicted activity of Cps3D and Cps7D.

Similarly, previously reported ^13^C and ^1^H spectra of the capsule polymers harvested from bacterial cultures of A. pleuropneumoniae serotype 7 (46) and serotype 3 (45) as well as from *N. meningitidis* serogroup H (41), which expresses the same polymer backbone as A. pleuropneumoniae serotype 3 (see Fig. S2), exactly match the ^1^H-^13^C correlations obtained by ^1^H, ^13^C HSQC for the polymers generated by Cps7D (Fig. 3d) and Cps3D (Fig. 3e), proving that both polymerases possessed their predicted activity.

Nevertheless, an additional set of signals slightly deviating from the published reference spectra was detected for both C1 and C1′ in the Cps7D product (Fig. 3d, red arrows), and, in addition to the major anomeric signal (76%), two additional minor anomeric signals (14% and 10%) could be detected in the spectrum of the Cps3D product (Fig. 3e, black, red, and magenta arrows, respectively).

We hypothesized that the additional signals resulted from the fact that the commercially available CDP-glycerol used in the Cps7D and Cps3D reactions was a racemic mixture containing both sn-glycerol-1-phosphate (C2′ of glycerol has S chirality) and sn-glycerol-3-phosphate (C2′ has R chirality; for nomenclature, see reference 40). Clearly, both enantiomers are substrates for the enzymes as shown by the complete consumption of CDP-glycerol in the HPLC assay (Fig. 2e and f). This is noteworthy, since, *in vivo*, both CDP-glycerol and the A. pleuropneumoniae serotype 7 capsule polymer have been reported to be enantiopure, consisting exclusively of sn-glycerol-3-phosphate (40, 46) (the stereochemistry of the A. pleuropneumoniae serotype 3 capsule polymer has not been investigated yet).

Consequently, we performed a comprehensive two-dimensional (2D) NMR analysis of the glycosidic linkage generated by Cps7D and Cps3D to elucidate if the nonnatural enantiomer (sn-glycerol-1-phosphate) is incorporated into the polymer and yields the observed additional signals.

### Stereochemistry of the polymer generated by Cps7D.

**FIG 4:**
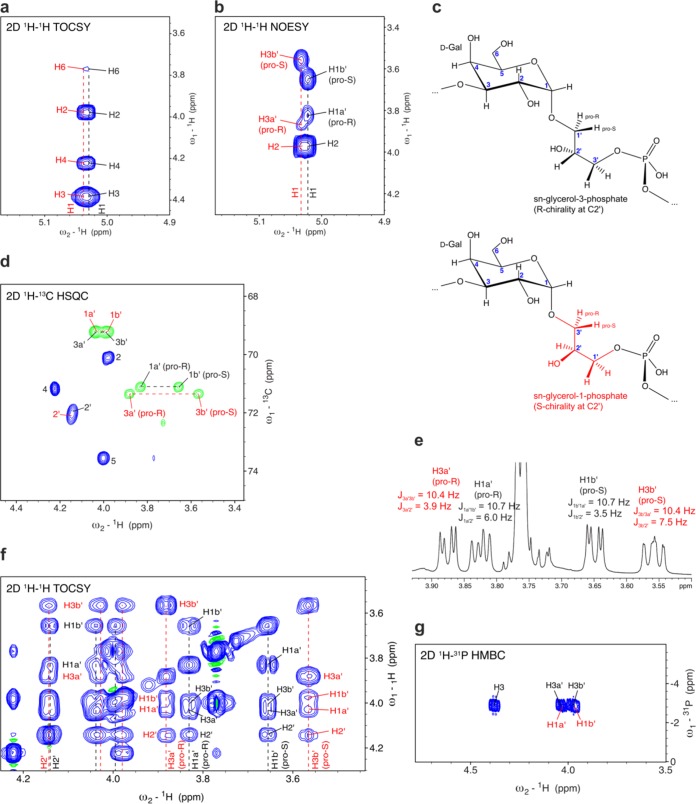
Stereochemistry of the polymer generated by Cps7D. The signals indicated in black and red are from spin systems containing sn-glycerol-3-phosphate and sn-glycerol-1-phosphate, respectively. (a) The 2D TOCSY correlations in the anomeric region demonstrate that the two spin systems are highly similar with regard to the galactose moiety. (b) The 2D NOESY correlations indicate differences between the two spin systems regarding the linkage to glycerol. (c) Chemical structures of the Cps7D products containing sn-glycerol-3-phosphate (black structure; C2′ has R chirality) and sn-glycerol-1-phosphate (red structure; C2′ has S chirality). The black structure is identical to the backbone of the capsule polymer expressed by A. pleuropneumoniae serotype 7. (d) ^1^H, ^13^C HSQC. Correlations corresponding to the CH and CH_2_ groups are shown in blue and in green, respectively. (e) The stereochemistry of the glycerol was determined from an ^1^H NMR spectrum according to a method described by Beynon et al., who determined for the R chirality at C2′ that ^3^J_H1′(pro-R)H2′_ must be as large as or larger than ^3^J_H1′(pro-S)H2′_, whereas for the S chirality at C2′, ^3^J_H3′(pro-R)H2′_ must be as small as or smaller than ^3^J_H3′(pro-S)H2′_ (46, 50). (f) Section of the 2D TOCSY data showing separate spin systems for sn-glycerol-3-phosphate (black) and sn-glycerol-1-phosphate (red). (g) ^1^H-^31^P HMBC data confirming the linkages between galactose, glycerol, and phosphate.

As expected, the 2D total correlation spectroscopy (TOCSY) spectrum of the Cps7D polymer (Fig. 4a) showed almost undistinguishable correlations for the two anomeric signals (5.04 ppm/101.5 ppm and 5.03 ppm/101.1 ppm) observed in the ^1^H, ^13^C HSQC (Fig. 3d, see C1 and red arrow), but their correlations in a 2D nuclear Overhauser effect spectroscopy (NOESY) spectrum were different (Fig. 4b). This confirmed our hypothesis that the galactose (Gal) spin systems were similar or identical, whereas the glycerol spin systems to which they are linked to were different.

In the 2D NOESY analysis (Fig. 4b), the spin system with black labels showed the NOE values to be 3.65 ppm (strong) and 3.82 ppm (weak), which were assigned to H1a′ and H1b′ of sn-glycerol-3-phosphate (Fig. 4c, black structure), matching the values previously reported for the natural polymer (46). Their corresponding correlations were isolated in the ^13^C-HSQC spectrum at 3.83 ppm/71.1 ppm and 3.65 ppm/71.1 ppm (Fig. 4d). The NOE cross-peak corresponding to 3.65 ppm was much more intense and could be assigned to the pro-S proton at C1′. The chirality at C2′ was found to be R, based on the scalar couplings observed on the isolated pro-S and pro-R protons attached to C1′ of glycerol. The coupling constant ^3^J_H1′(pro-R)H2′_ value was found to be 6.0 Hz, a value larger than the value of 3.5 Hz determined for ^3^J_H1′(pro-S)H2′_ (Fig. 4e), and fits with the conformation shown in Fig. 4c (black structure), in which H1a′ (pro-R) is in *trans* to H2′. This agrees with Beynon et al., who determined for the R chirality at C2′ that the ^3^J_H1′(pro-R)H2′_ value must be equal to or larger than the ^3^J_H1′(pro-S)H2′_ value, considering various rotamers (46, 50).

The second anomeric signal of galactose, indicated with red labels, shows NOE correlations to 3.56 ppm (strong) and 3.87 ppm (weak) (Fig. 4b), which were assigned to H3a′ and H3b′ of sn-glycerol-1-phosphate, distinguishing itself from sn-glycerol-3-phosphate solely by the chirality at C2′ (Fig. 4c, red structure). By definition, the carbon numbering is inverted. Thus, their correlations in the ^13^C-HSQC spectrum at 3.56 ppm/71.4 ppm and 3.88 ppm/71.4 ppm are in the same region as the H1a′ and H1b′ signals of the sn-glycerol-3-phosphate (Fig. 4d). The stronger NOE to 3.56 ppm defines the pro-S proton at C3′. The fact that the coupling constant ^3^J_H3′(pro-R)H2′_ = 3.9 Hz is smaller than ^3^J_H3′(pro-S)H2′_ = 7.5 Hz (Fig. 4e) fits with the conformation with S chirality at C2′ shown in Fig. 4c (red structure), in which the H1b′ (pro-S) is in *trans* to H2′, again agreeing with Beynon et al., who determined that ^3^J_H3′(pro-R)H2′_ must be equal to or smaller than ^3^J_H3′(pro-S)H2′_ (46, 50).

The two spin systems of the glycerol are separated in the 2D TOCSY spectrum (Fig. 4f), which shows that their methylene group (H1′ for R chirality and H3′ of S chirality) signals are distinguishable, whereas their resonances with respect to H2′ and the other CH_2_ group with the phosphate attached are almost identical. The two signals of phosphate-attached CH_2_ of glycerol at ~4.04 ppm/69.2 ppm and ~3.98/69.2 ppm in the ^13^C-HSQC spectrum contain overlapping signals of H3a′ and H3b′ for the R chirality (black label) and H1a′ and H1b′ for the S chirality (red label). The 2D TOCSY data reveal that H3a′ and H3b′ of the R form correspond to chemical shifts of 4.04 and 4.00 ppm, whereas H1a′ and H1b′ of the S form correspond to chemical shifts of 4.03 and 3.98 ppm. Both of these methylene groups show correlations to the phosphate in the ^1^H-^31^P heteronuclear multiple bond correlation (HMBC) spectrum (Fig. 4g), confirming the structures shown in Fig. 4c. In summary, the detailed NMR analysis of the Cps7D product confirmed that the observed second set of signals exclusively resulted from the incorporation of the nonnatural substrate sn-glycerol-1-phosphate.

### Stereochemistry of the polymer generated by Cps3D.

**FIG 5:**
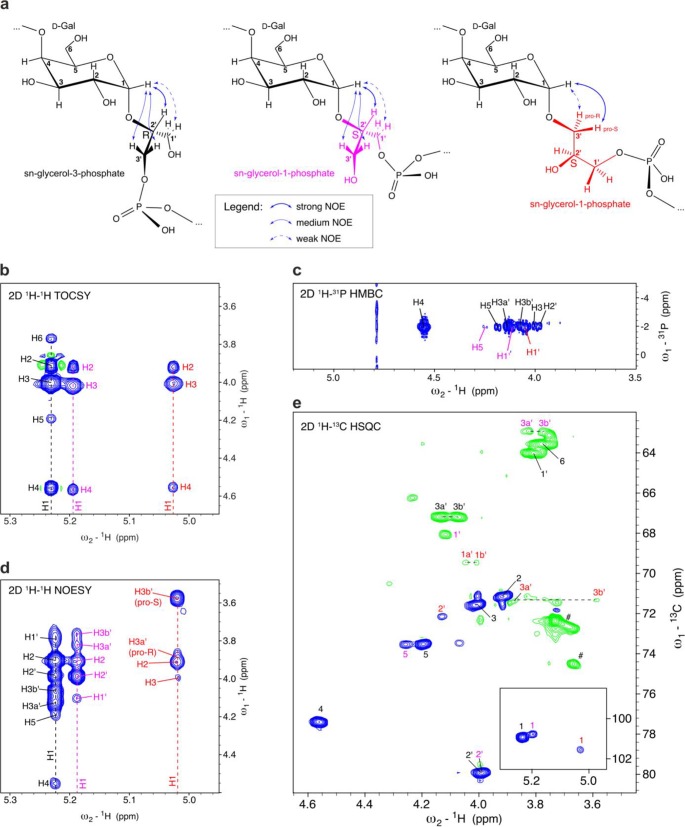
Stereochemistry of the polymer generated by Cps3D. (a) Structures derived for the spin systems belonging to the major anomeric signal (black) and the two minor anomeric signals (magenta and red). The strengths of the NOEs between the anomeric proton and various protons of the glycerol are indicated by blue arrows (see key). (b) Section of the anomeric region of a 2D TOCSY experiment, demonstrating that the galactose moieties in all three spin systems are linked to a phosphate at C4. (c) Corresponding ^1^H-^31^P HMBC. (d) 2D NOESY data indicating that the galactose moieties are linked to C2′ of the glycerol in the black and magenta spin system, whereas the linkage is placed at C1′ in the red spin system. (e) ^1^H, ^13^C HSQC correlations of all three spin systems. Signals labeled with a crosshatch symbol (#) are due to process-related impurities which were absent in a second batch of Cps3D product (data not shown).

For the Cps3D product, our first aim was to clearly confirm the assignment shown for the dominant spin system in Fig. 3e. The strong anomeric signal at 5.23 ppm belonging to this spin system (black arrow in Fig. 3e, black structure in Fig. 5a) shows correlations in a 2D TOCSY spectrum to five other protons (H2 and H3, H4 and H5, and H6 of galactose), among which one was significantly shifted downfield (Fig. 5b, black labels). Establishing the H1 and H2, H2 and H3, and H3 and H4 correlations in a 2D COSY spectrum (data not shown) showed that the downfield-shifted resonance can be assigned to H4, which showed a correlation to ^31^P in a ^1^H-^31^P HMBC spectrum, indicating a linkage between phosphate and C4 (Fig. 5c). A strong NOE between the anomeric proton and H2′ confirmed the linkage to C2′ of glycerol (Fig. 5d) and thus the identity to the backbone of the capsule polymer expressed by A. pleuropneumoniae serotype 3 (45) (Fig. 5a, black structure). The chirality at the glycerol carbon C2′, not determined previously (45), is derived in the following sections.

At lower contour levels, signals of two other galactose spin systems became visible (Fig. 5e, marked with red and magenta labels). The TOCSY correlations are practically identical to those seen with the dominant spin system (Fig. 5b), including the downfield-shifted H4 resonance, suggesting that they all belong to a galactose with a phosphate substitution at C4.

The difference between the black and the magenta spin systems becomes apparent by visualizing the glycosidic linkage as shown in Fig. 5a. Assuming that both repeating units adopt the most likely populated conformation, in which the exo-anomeric effect governs the phi-psi angles of the glycosidic linkage, the C3′ points toward the reader. The strong NOE between galactose H1 and H2′ observed for both spin systems (Fig. 5a and d) suggests that H2′ is orientated upwards, coming close to galactose C1 and O5. The chirality at C2′ is determined by the phosphate; if the phosphate is attached to C3′ in front, C2′ has R chirality (sn-glycerol-3-phosphate, black structure), and if the phosphate is attached to C1′, C2′ has S chirality (sn-glycerol-1-phosphate, magenta structure). The glycerol CH_2_ group with the shortest distance to galactose H1 is the one in front at C3′. Consistent with these considerations, the strongest NOEs with respect to 4.13 and 4.06 ppm in the black spin system resulted from H3a′ and H3b′, which also showed correlations to ^31^P (Fig. 5c), confirming that the black spin system had R chirality at C2′ and contained sn-glycerol-3-phosphate. This is further supported by the effect of the phosphate on the ^13^C and ^1^H chemical shifts; C3′ was shifted farther downfield than C1′ (Fig. 5e), and both H3a′ and H3b′ appeared to have been shifted farther downfield than H1′ (Fig. 5d). In the case of the magenta spin system, the strongest NOEs were seen in the upfield resonances of 3.82 and 3.77 ppm, which in turn did not show correlations to ^31^P, indicating S chirality. Only a weak NOE was seen for the H1′ signal, which was shifted downfield and showed correlations to ^31^P and thus is linked to phosphate, confirming a C2′ with S chirality (sn-glycerol-1-phosphate).

While the black-labeled and magenta-labeled spin systems showed a very strong NOE at 3.98/3.99 ppm (corresponding to H2′ of the glycerol and thus indicating a linkage to C2′), the red spin system showed a strong NOE at 3.58 ppm (Fig. 5a and d), which originated from a CH_2_ group (Fig. 5e). Interestingly, the resonances of the red spin system showed striking similarity to those seen with the sn-glycerol-1-phosphate-containing repeating units generated by Cps7D (Fig. 5 and 4, red labels). Thus, the resonance at 3.58 ppm was assigned to the pro-S proton at C3′ of sn-glycerol-1-phosphate. This assignment suggests that the inverted stereochemistry of the nonnatural substrate sn-glycerol-1-phosphate forces Cps3D to misplace the glycosidic linkage and connect C1 of galactose to C1′ of glycerol instead to C2′. However, as shown by 2D TOCSY (Fig. 5b), the phosphodiester bond is still correctly placed at C4 of the galactose rather than at C3 as reported for the product of Cps7D (compare Fig. 5a and 4a, red structures). In summary, the NMR study data presented above clearly demonstrate that the observed heterogeneity in the Cps3D product exclusively originated from incorporation of the nonnatural substrate sn-glycerol-1-phosphate.

### Role of the tetratricopeptide domain.

**FIG 6:**
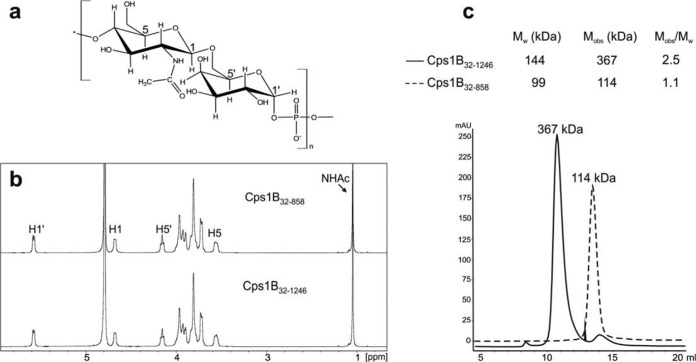
Truncation studies of Cps1B. (a) Chemical structure of the Cps1B product. (b) ^1^H NMR spectra demonstrating the identity of the polymers generated by Cps1B_32–1246_ and Cps1B_32–858_. (c) Elution profiles of an analytic size exclusion chromatography experiment. M_W_, theoretical molecular mass of the proteins in a monomeric state; M_obs_, observed molecular mass in the experiment.

Many TagF-like polymerases were predicted to contain a domain that can be modeled onto templates rich in tetratricopeptide repeats (TPRs) (Fig. 1c and d; see also Fig. S3). Since TPRs are known for mediating protein-protein interactions (59), we hypothesized that the predicted TPR domains do not participate in the catalytic activity of the polymerases. To confirm this hypothesis, we truncated the TPR domain in Cps1B and Cps7D as representative candidates for the GT-A/TagF-like and the TagF-like/GT-B architectures, respectively. Unfortunately, the resulting Cps7D construct showed low expression levels and could not be purified (data not shown). Since Cps1B, like many group 2 capsule polymerases (23, 29), was shown to be expressed as an N-terminal truncation lacking the first 31 amino acids (Fig. S7), the TPR truncation was introduced in Cps1B_32–1246_, yielding Cps1B_32–858_. Cps1B_32–858_ could be expressed and purified, maintained its activity in the HPLC-AEC assay (data not shown), and the ^1^H NMR spectra obtained for the polymer synthesized by Cps1B_32–1246_ and Cps1B_32–858_ were identical (Fig. 6a and b). However, it is of note that in analytic size exclusion chromatography, Cps1B_32–1246_ eluted with an apparent molecular mass corresponding to a dimeric to trimeric assembly, whereas Cps1B_32–858_-His_6_ appeared to be monomeric, indicating that the TPR domain mediates oligomerization of Cps1B (Fig. 6c).

### Mutational studies of TagF-like polymerases.

**FIG 7:**
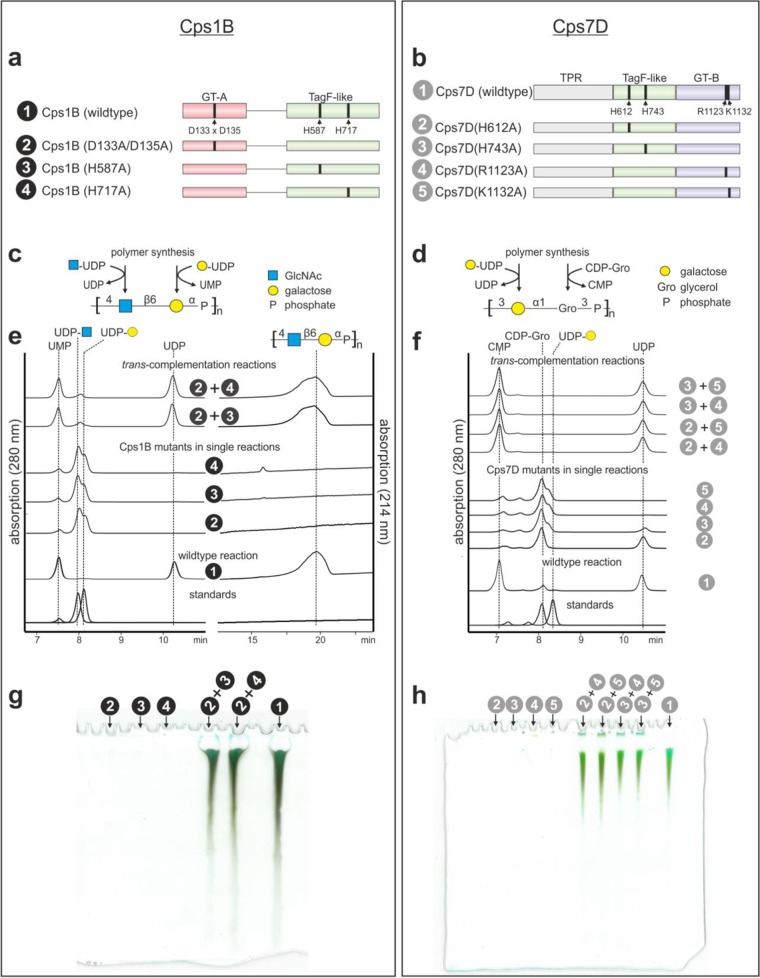
Site-directed mutagenesis studies of Cps1B (left panel) and Cps7D (right panel). (a and b) Schematic representation of Cps1B (a) and Cps7D (b) showing conserved residues in each domain and the single-domain mutants indicated by black bars. (c and d) Cps1B (c) and Cps7D (d) reaction schemes. (e) HPLC-AEC analyses show that all single-domain mutants (constructs 2 to 4) were unable to produce polymer (214-nm channel). Small amounts of UMP/CMP detected in the reactions of Cps1B (e) and Cps7D (f) mutants were found in the controls (standards) as well, indicating that they resulted from spontaneous, enzyme-independent hydrolysis (280-nm channel). However, combining two single-domain mutants in *trans* restores donor substrate uptake and polymer synthesis, as documented by HPLC-AEC (214-nm channel) (e and f) and alcian blue/silver-stained PAGE (g and h).

The correctness of the PHYRE2 modeling was corroborated by the fact that amino acids known to have catalytic functions in TagF (H444, 584) (54), K4CP (D519xD521 motif) (57), and TarM (R326, K331) (58) (all residues involved in coordinating the pyrophosphate of the donor substrate) could be superimposed with identical residues in the predicted TagF-like, GT-A folded, and GT-B folded domains, respectively (Fig. 1e), even though the percentage of sequence identity observed between the predicted domains and their templates was low (Fig. S4 and S6). Moreover, multiple-sequence alignments performed using Clustal Omega (56) demonstrated these residues to be part of larger sequence motifs that appeared to be conserved in both the predicted domains and their corresponding templates (Fig. S5, S9, and S10). Focusing on Cps1B (expressed as Cps1B_32–858_-His_6_) and Cps7D (expressed as MBP-Cps7D-His_6_), all conserved positions were mutated to alanine to give the single-domain mutants Cps1B(D133A/D135A), Cps1B(H587A), and Cps1B(H717A) (Fig. 7a), as well as Cps7D(H612A), Cps7D(H743A), Cps7D(R1123A), and Cps7D(K1132A) (Fig. 7b). Mutant constructs were expressed in E. coli and subsequently purified at levels comparable to the wild-type levels. As expected, when activity was assayed using the HPLC-AEC assay (Fig. 7c to f) and PAGE (Fig. 7g and h), no polymer synthesis could be observed after 3 h of incubation (Fig. 7g and h) and the level of UMP/CMP detected in the reactions was comparable to those seen with the negative controls and could thus be attributed to spontaneous hydrolysis of the donor substrates (Fig. 7e and f, 280-nm channel). Interestingly, considerable levels of UDP, most likely due to enzyme-facilitated hydrolysis of UDP-galactose (UDP-Gal), could be detected in the presence of TagF-like domain mutants Cps7D(H612A) and Cps7D(H743A) (Fig. 7f, constructs 2 and 3), indicating that the GT-B folded domain was unaffected by the mutation in the TagF-like domain and corroborating its GT activity (hexose-1-phosphate transferase activity would release UMP). Assuming that each single-domain mutant should still contain one domain that remained functional, we performed *trans*-complementation reactions, combining the GT-A domain mutant and the TagF-like domain mutants of Cps1B as well as the TagF-like domain mutants and the GT-B domain mutants of Cps7D. Indeed, donor substrate consumption (Fig. 7e and f) and polymer synthesis (Fig. 7g and h) were restored to wild-type levels in all *trans*-complementation reactions, indicating that the two remaining unmodified domains were able to catalyze the reaction in *trans*.

10.1128/mBio.00641-18.9 FIG S9  Sequence alignment of all predicted N-terminal GT-A domains analyzed in this study, including the sequence of the K4CP template (UniProt: Q8L0V4) used for PHYRE2 modeling. Database references for all TagF-like polmyerase sequences are indicated in the Fig. S3 legend. Identical amino acids are shown in gray boxes, and aspartate residues of the conserved DxD motif are highlighted in red. The sequence alignment was performed with Clustal Omega (F. Sievers, A. Wilm, D. Dineen, T. J. Gibson, K. Karplus, W. Li, R. Lopez, H. McWilliam, M. Remmert, J. Söding, J. D. Thompson, and D. G. Higgins, Mol. Syst. Biol. **7:**539, 2011) on the UniProt website (http://www.uniprot.org/align/) (E. Boutet, D. Lieberherr, M. Tognolli, M. Schneider, and A. Bairoch, Methods Mol. Biol. **406:**89–112, 2007) and annotated using Jalview software (A. M. Waterhouse, J. B. Procter, D. M. A. Martin, M. Clamp, and G. J. Barton, Bioinformatics **25:**1189–1191, 2009). Download FIG S9, PDF file, 0.4 MB. Copyright © 2018 Litschko et al. 2018 Litschko et al. This content is distributed under the terms of the Creative Commons Attribution 4.0 International license.

10.1128/mBio.00641-18.10 FIG S10  Sequence alignment of all predicted C-terminal GT-B domains analyzed in this study, including the sequence of the TarM template of Staphylococcus aureus (UniProt: A0A0J9X257) used for PHYRE2 modeling. Database references for all TagF-like polymerase sequences are indicated in the Fig. S3 legend. Identical amino acids are shown in gray boxes, and the conserved arginine and lysine residues are highlighted in red. The sequence alignment was performed with Clustal Omega (F. Sievers, A. Wilm, D. Dineen, T. J. Gibson, K. Karplus, W. Li, R. Lopez, H. McWilliam, M. Remmert, J. Söding, J. D. Thompson, and D. G. Higgins, Mol. Syst. Biol. **7:**539, 2011) on the UniProt website (http://www.uniprot.org/align/) (E. Boutet, D. Lieberherr, M. Tognolli, M. Schneider, and A. Bairoch, Methods Mol. Biol. **406:**89–112, 2007) and annotated using Jalview software (A. M. Waterhouse, J. B. Procter, D. M. A. Martin, M. Clamp, and G. J. Barton, Bioinformatics **25:**1189–1191, 2009). Download FIG S10, PDF file, 0.7 MB. Copyright © 2018 Litschko et al. 2018 Litschko et al. This content is distributed under the terms of the Creative Commons Attribution 4.0 International license.

## DISCUSSION

This report presents the identification of a novel family of bacterial multidomain enzymes termed "TagF-like capsule polymerases" that generate complex phosphate-containing group 2 capsule polymers. PHYRE2 (53) homology modeling, complemented by experimental data, suggests that the TagF-like domain, conserved in all TagF-like polymerases, catalyzes the transfer of hexose-phosphate and glycerol-phosphate residues, whereas adjacent GT-A and GT-B folded domains transferred hexose residues with inverted stereochemistry (β-glycosidic linkages) or retained stereochemistry (α-glycosidic linkages), respectively. Mutations in the catalytic core, selected based on the template structures of TagF (54), K4CP (GT-A) (57) and TarM (GT-B) (58), abolished activity, strongly indicating the correctness of the modeling results. Importantly, *trans*-complementation restored activity to wild-type levels, corroborating that each domain is independent from the other with regard to activity and folding.

Using size exclusion chromatography and ^1^H NMR, we showed that the TPR (tetratricopeptide repeat) domain, predicted at the N terminus or C terminus of the majority of TagF-like polymerases, mediates oligomerization in Cps1B but is not required for enzymatic activity. Unfortunately, attempts to truncate the N-terminal TPR domain in Cps7D resulted in abolishment of expression, suggesting that the TPR domain may play a more important role with regard to protein stability and/or folding in polymerases with TagF-like/GT-B architecture. Given the considerable size (~400 amino acids) of the predicted TPR domains, it is likely that they adopt other functions in the *in vivo* context, such as mediating interactions with the ABC transporter of the capsule biosynthesis complex, with polymer-modifying enzymes, or with sugar/glycerol-activating enzymes (1).

Bacteria expressing TagF-like polymerases were largely identified through analysis of published capsule structures (only Cps12B, Cps4B, and Ccs2 could be identified by BLAST searches performed using the CslB sequence as the query). Those bacterial species are as diverse as the meningitis-causing agent N. meningitidis and uropathogenic E. coli K2 (both human pathogens) and the animal respiratory pathogens A. pleuropneumoniae and B. trehalosi. Consequently, the polymer structures generated by TagF-like polymerases as well as the sequence identity between the enzymes can vary considerably. The neisserial polymerases CslB and Cshc, which generate a trimeric poly(oligosaccharide phosphate) and a dimeric poly(glycosylpolyol phosphate) repeating unit (29, 41), respectively, share only 14% sequence identity, whereas enzymes generating identical structures can share up to 70% sequence identity. Consistent with the fact that the TagF-like domain is most ambiguous (it is able to transfer both glycerol-phosphate and hexose-phosphate residues), the percentages of sequence identity between TagF-like domains from different strains represent a large range of diversity (from 15% to 93%), whereas the GT-A and GT-B folded domains share 30% to 70% and 40 to 90% identity, respectively. To retrospectively assess the distribution of TagF-like polymerases, BLAST searches were performed using the amino acid sequences of newly identified Cps1B and Cps7D, yielding homologues with sequence identities ranging from 35% to 65% in a variety of pathogens such as Actinobacillus suis, Campylobacter jejuni, Campylobacter coli, Mannheimia varigena, Neisseria mucosa, and Yersinia enterocolitica. This finding suggests that TagF-like polymerases also play a role in a multitude of bacterial species whose capsule structures have not been investigated yet.

Capsule polymers are utilized to produce efficient glycoconjugate vaccines, in which polymer fragments are covalently coupled to carrier proteins to elicit T-cell responses and immunologic memory (60). Immunizations with glycoconjugates containing capsule polymers from, e.g., H. influenzae, S. pneumoniae, and N. meningitidis, have been proven highly successful in preventing infectious diseases in humans (61), and experimental glycoconjugate vaccines against animal pathogens, e.g., A. pleuropneumoniae serotype 1, have been documented to produce strong immune responses (62, 63). Unfortunately, the biohazards and costs associated with glycoconjugate production are key barriers for broad market launch (64). Current protocols require isolation of capsule polymers from large-scale fermentation of pathogenic bacteria. This step essentially depends on the high-tech infrastructure of modern production plants, which require the highest biosafety standards (61), making glycoconjugate vaccines expensive; not sufficiently accessible to low-income countries; and, with regard to animal husbandry, where they could reduce/avoid the exuberant use of antibiotics, not sufficiently cost-effective (65, 66). We recently showed that enzymatic synthesis of the capsule polymer from *N. meningitidis* serogroup X provides a simple, biohazard-free, and thus potentially more cost-effective means for the production of a functional glycoconjugate vaccine (25, 27, 67). The identification of the TagF-like polymerase family vastly increases the number of enzymes and thus the number of capsule polymers available for the development of glycoconjugate vaccines against other human and even animal pathogens.

The poly(glycosylpolyol phosphate) structures generated by the TagF-like/GT-B folded polymerases have in the past been described as teichoic acid-like polymers due to their similarity to WTA type II (48, 50), a common polymer of the cell envelope of Gram-positive bacteria. Unfortunately, little is known about WTA type II biosynthesis and further studies are needed to investigate if homologues of TagF-like polymerases play a role (39, 40). Nonetheless, TagF (54) and TarM (58), the template structures for modeling the TagF-like domain and the GT-B domain, polymerize and modify wall teichoic acid type I, respectively. Despite the fact that TarM and TagF are expressed in Gram-positive bacteria and act as separate polypeptides, they share conserved amino acids and, in case of TagF, even all active-site motifs (54) with the domains of the TagF-like polymerases. It is thus tempting to speculate that they were acquired by Gram-negative group 2 capsule-expressing bacteria through horizontal gene transfer or vice versa. Further research is needed to elucidate the evolutionary relationship between WTA type I and group 2 capsule biosynthesis.

The NMR characterization of the Cps7D and Cps3D products clearly demonstrated that both enzymes consumed sn-glycerol-3-phosphate and sn-glycerol-1-phosphate from the racemic CDP-glycerol mixture, whereas A. pleuropneumoniae serotype 3 and 7 capsules exclusively contain sn-glycerol-3-phosphate (45, 46). sn-Glycerol-1-phosphate is commonly found in lipoteichoic acid and is supplied from the donor substrate phosphatidylglycerol on the outer side of the Gram-positive cell membrane, whereas the sn-glycerol-3-phosphate found in WTA is usually provided by CDP-glycerol in the cytosol (40). In agreement with that, group 2 capsule biosynthesis takes place on the cytoplasmic side of the inner membrane and a glycerol-3-phosphate cytidylyltransferase has been predicted in region 2 of the A. pleuropneumoniae serotype 3 and 7 capsule gene cluster (10). It is tempting to speculate that the lack of selective pressure for sn-glycerol-3-phosphate resulting from the absence of sn-glycerol-1-phosphate in the cytosol is the reason for the observed substrate ambiguity of Cps7D and Cps3D.

In contrast to all other polymerases analyzed in this study, the candidates from B. trehalosi (B. trehalosi 188 [Bt188], Bt189, Bt192, and BtY31 [68, 69]) (see Fig. S2 and Fig. S3 in the supplemental material) were identified in genomes of nonserotyped strains. The capsules of three of the four known *B. trehalosi* serotypes have been analyzed by NMR in the past, and all of those studies reported a poly(glycoslypolyol phosphate) structure (50–52). In agreement with these findings, the polymerases encoded in the Bt188, Bt189, and Bt192 genomes adopt the TagF-like/GT-B architecture. Interestingly, homology modeling for the polymerase encoded in the BtY31 genome predicts a GT-A/TagF-like fold, suggesting that BtY31 expresses a poly(oligosaccharide phosphate) capsule. Although a structural analysis of the BtY31 capsule polymer will be required for confirmation, this finding already highlights that the knowledge presented here about the TagF-like polymerase family can be exploited to predict the composition of unknown capsule structures, providing a starting point for structural analyses.

In summary, the identification of the TagF-like capsule polymerase family (i) reveals a new class of polymerases known to be involved in group 2 capsule biosynthesis, (ii) represents a new source for synthetic biomaterials, (iii) allows conclusions to be drawn with respect to the relationship between the protein repertoire and capsule structure, and (iv) potentially provides a link between cell wall biosynthesis in Gram-positive and that in Gram-negative bacteria.

## MATERIALS AND METHODS

### Bioinformatics.

Homologues of CslB were identified by protein BLAST searches using the CslB amino acid sequence as the query and the BLASTP algorithm (32), homology modeling was performed using the intensive mode of the structure prediction tool PHYRE2 (53), and sequence alignments were performed using Clustal Omega (56).

### General cloning.

The generation of plasmid *p*ΔN37-cslB-His_6_ (tac) encoding CslB_38–874_-His_6_ was described previously (29). *cps1B* (GenBank accession number KY798410), *cps3D* (GenBank accession number KY807157), *cps7D* (GenBank accession number ACE62291.1), *cps12B* (GenBank accession number AY496881.1), and *ccs2* (GenBank accession number AEC50903.1) were amplified by PCR from heat-inactivated bacterial lysates using the primers given in Tables 3 and 4. The resulting PCR products amplified from *cps1B*, *cps3D*, *cps7D*, and *cps12B* were cloned via the indicated restriction sites (Tables 3 and 4) into plasmid *p*MBP-csxA-His_6_ (tac) (22), replacing the *csxA* sequence and resulting in *p*MBP-cps1B-His_6_, *p*MBP-cps3D-His_6_, *p*MBP-cps7D-His_6_, and *p*MBP-cps12B-His_6_, respectively. *ccs2* was cloned into *p*ΔN37-cslB-His_6_ (tac) (29). Single-amino-acid mutations and truncations were introduced using a Q5 site-directed mutagenesis kit (New England Biolabs) according to the manufacturer’s guidelines. Plasmid *p*cps1B_32–858_-His_6_ (tac) was cloned in two steps. First, plasmid *p*cps1B_32–1246_-His_6_ (tac) was generated with primers CL102/CL104 and *p*MBP-cps1B-His_6_ as the template. Subsequently, plasmid *p*cps1B_32–858_-His_6_ (tac) was generated with primers CL128/CL129 and *p*cps1B_32–858_-His_6_ as the template. Single amino acid mutations were generated using primers shown in Table 4 and *p*cps1B_32–858_-His_6_ (tac) or *p*MBP-cps7D-His_6_ as the template.

**TABLE 2:** Strains, polymerases, and corresponding accession numbers used in this study

Strain	Polymerase	GenBank accession no.
Actinobacillus pleuropneumoniae 4074	Cps1B	KY798410
Actinobacillus pleuropneumoniae S1421	Cps3D	KY807157
Actinobacillus pleuropneumoniae AP76	Cps7D	ACE62291.1
Actinobacillus pleuropneumoniae 8329	Cps12B	AY496881.1
Haemophilus influenzae ATCC 9007	Ccs2	AEC50903.1

**TABLE 3:** Plasmids used in this study^a^

Plasmid	Recombinant construct	Molecular mass (kDa)	Primer	Restriction sites
*p*MBP-cps1B-His	MBP-Cps1B-His	190	CL57/CL59	BamHI/AvrII
*p*MBP-cps3D-His	MBP-Cps3D-His	177	CL147/CL148	BamHI/XhoI
*p*MBP-cps7D-His	MBP-Cps7D-His	192	CL74/CL94	BamHI/AvrII
*p*MBP-cps12B-His	MBP-Cps12B-His	146	CL33/CL56	BamHI/XhoI
*p*ccs2-His	Ccs2-His	144	CL40/CL39	NdeI/XhoI
*p*Cps1B_32–1246_-His	Cps1B_32–1246_-His	144	CL102/CL104	
*p*Cps1B_32–858_-His	pCps1B_32–858_-His	99	CL128/CL129	

a MBP, maltose binding protein.

**TABLE 4:** Primers used in this study

Primer designation or category^a^	Primer sequence^b^
CL57	5′-CCATAGGGATCCAATAAAGTAAAACGTAAATTTAG-3′
CL59	5′-CTTTTACCTAGGAACGCCCAACTTAATTAACATTAGTGGTGGTGGTGGTGGTGCTCGAGGATGAATTTTTCAAAAAAGATAG-3′
CL147	5′-CCATAGGGATCCTTAATAAACAACGAGAATG-3′
CL148	5′-GGTGCTCGAGTTTAGTATTTTCGTTAAATTC-3′
CL74	5′-CCATAGGGATCCAAGAAAAAATTTTATAAAGC-3′
CL94	5′-CTTTTACCTAGGAACGCCCAACTTAATTAACATTAGTGGTGGTGGTGGTGGTGCTCGAGAATAACATTATAAAATCTATTAATTG-3′
CL33	5′-CCATAGGGATCCAATAAAATTAGTA-3′
CL56	5′-GGTGCTCGAGGTTTATATTTCTTTTTGG-3′
CL40	5′-GCATCTCATATGAGCAAAATCAATAGAAAACTTAAGAAAC-3′
CL39	5′-GGTGCTCGAGTGAAAGTAAATCGGCTAATTTTAATTG-3′
Primers used for generating truncated Cps1B constructs	
CL102	5′-AAACATTTACCTGTTAAATATGAAG-3′
CL104	5′-CATGGACTATGGTCCTTG-3′
CL128	5′-ATCCGGCATATCTAAGTTAATAATAG-3′
CL129	5′-CTCGAGCACCACC-3′
Primer pairs used for generating the underlined mutations in *cps1B*	
D133A/D135A	
CL162	5′-TTACCTTTATTGCGCCAGCGGATTTTCTTAG-3′
CL163	5′-CCCATTCTGTTTGTACGTATTTTAGTCC-3′
H743A	
CL137	5′-TAAAGATGATTTATCTCAATGGTTC-3′
CL136	5′-GTTATACCCGCCTGTAAA-3′
H717A	
CL164	5′-CCATATTTAAATGAGTTTAACATCCCC-3′
CL165	5′-TTCAATATTAGGCGCTGGTGCAAAAATAAC-3′
Primer pairs used for generating the underlined mutations in *cps7D*	
H612A	
CL177	5′-TTAAAACATTAGGAAGAGATATGGAG-3′
CL176	5′-ATGGAGTTCCCGCCCATGTAC-3′
H743A	
CL179	5′-AAGCATTATCCAAAATTAATCTAG-3′
CL178	5′-CCTGAAGTAATGACGCCCCTCTAA-3′
R1123A	
CL181	5′-GAGAAGGACCACGCTAAGT-3′
CL180	5′-TATTGATAGCGCACCTATTGTT-3′
K1132A	
CL160	5′-GCTAAGTTAATTAATAGTTTTGC-3′
CL159	5′-GTGGTCCGCCTCTATT-3′

a Primers not otherwise defined were used for generating the constructs shown in Table 3.

b Restriction sites are underlined.

### Expression and purification of recombinant proteins.

Expression and purification of recombinant constructs were performed as previously described (29). Briefly, E. coli M15[pREP4] cells were transformed with the plasmids shown in Table 3 and protein expression was induced with 1 mM IPTG (isopropyl-β-d-thiogalactopyranoside) when 500 ml of expression culture (Power Broth medium) reached an optical density at 600 nm (OD_600_) of 0.6 to 1.0. Expression was performed at 15°C and 200 rpm for 21 h. After the cells were harvested by centrifugation, the cell pellet was resuspended in lysis buffer (50 mM Tris [pH 8.0], 500 mM NaCl, 2 mM dithiothreitol [DTT], 0.2 mg/ml DNase [Roche], 0.1 mg/ml RNaseA [Roche], 0.1 mg/ml lysozyme [Serva], and EDTA-free protease inhibitor [Complete EDTA-free; Roche]) and the cell suspension was subjected to sonification (Branson Digital Sonifier) (50% amplitude; sonification was performed 8 times for 30 s each time with cooling on ice between the sonification stops). Recombinant His_6_-tagged proteins were enriched by immobilized metal ion affinity chromatography and eluted using an imidazole gradient (25 to 500 mM imidazole over 20 min). Protein-containing fractions were pooled and applied to a size exclusion chromatography column (Superdex 200 10/300 GL [GE Healthcare] or HiPrep 26/10 Desalting [GE Healthcare]) for further purification and/or buffer exchange, after which aliquots were snap-frozen in liquid nitrogen and stored at −80°C. Analytic size exclusion chromatography was performed using a Superdex 200 10/300 Gl column (GE Healthcare) and a gel filtration marker kit for protein molecular weights 12,000 to 200,000 (Sigma) according to the manufacturer’s guidelines.

### Enzymatic reactions and analysis via HPLC and polyacrylamide gel electrophoresis (PAGE).

Enzymatic reactions were carried out with 0.1 to 0.3 nmol of purified protein in a total volume of 75 µl of assay buffer (20 mM Tris [pH 8.0], 1 mM DTT, 10 mM MgCl_2_, 6 to 10 mM donor sugar). UDP-GlcNAc (Carbosynth), UDP-Gal (Carbosynth), and CDP-glycerol (racemic; from Sigma-Aldrich) were used as donor substrates as indicated in Fig. 2. Reaction mixtures containing wild-type proteins were incubated for 24 h at 37°C, while mutant constructs were incubated for 3 h at 37°C to minimize spontaneous hydrolysis of donor substrates. HPLC-based anion exchange chromatography (HPLC-AEC) was performed on a Prominence UFLC-XR system (Shimadzu) equipped with a CarboPac PA-100 column (Dionex) (2 by 250 mm). Samples were separated as described in reference 29, with the adjustment that 20 mM Tris (pH 8.0) and 20 mM Tris (pH 8.0) plus 1 M NaCl were used as mobile phases M1 and M2, respectively. Six microliters of the samples was loaded for the detection of nucleotides at 280 nm and 50 µl for the detection of capsule polymer at 214 nm. Nucleotides were separated using a linear elution gradient of 0% to 30% M2 over 11 min. Polymers were separated using an elution gradient consisting of a −2 curved gradient of 0% to 30% M2 over 4 min followed by a linear gradient of 30% to 84% M2 over 33 min. Chromatography was conducted at 0.6 ml/min with a column temperature of 50°C. Four-microliter volumes of Cps3D and Cps7D reaction samples mixed with 4 µl of a 2 M sucrose solution were used for separation on high-percentage (15%) PAGE and visualized by a combined alcian blue/silver staining procedure as described in reference 70.

SDS polyacrylamide gel electrophoresis (SDS-PAGE) was performed as described in reference 29.

### Upscaling of *in vitro* polymer synthesis and subsequent purification.

For *in vitro* synthesis of 5 to 12 mg of polymer, protein (1 to 25 nmol) was incubated overnight at 37°C in reaction buffer (20 mM Tris [pH 8.0], 10 mM MgCl_2_, 1 mM DTT) with a 6 to 10 mM concentration of the activated substrates in a total volume of 5 to 10 ml. *In vitro*-synthesized polymer was purified by anion exchange chromatography (AEC) using a MonoQ HR10/100 Gl column (GE Healthcare) and a linear NaCl gradient (over 41 min) starting at 0 to 1 M NaCl at a flow rate of 1 ml/min. Polymer-containing fractions were pooled, dialyzed against water (ZelluTrans; Roth) (1,000 molecular weight cutoff [MWCO]), and freeze-dried for further analysis.

### NMR analysis.

The NMR spectra shown in Fig. 3a to c and described in Table 1 were determined using a BrukerAvance III 400 MHz spectrometer equipped with a 5-mm broadband probe (Bruker). ^1^H NMR spectra were collected at 298 K with 32,000 data points over a 10-ppm spectral width, accumulating an appropriate number of scans. The spectra were generally weighted with 0.2-Hz line broadening and were subjected to Fourier transformation. The transmitter was set at the water frequency which was used as the reference signal (4.79 ppm). All the ^1^H spectra were obtained in a quantitative manner using the total recycle time to ensure a full recovery of each signal (5 × longitudinal relaxation time *T*_1_). ^13^C NMR spectra were recorded at 100.6 MHz and 298 K, with 32,000 data points over a 200-ppm spectral width, accumulating an appropriate number of scans. The spectra were generally weighted with 1.0-Hz line broadening and were subjected to Fourier transformation. The transmitter was set at the acetone frequency, as an external calibration, which was used as the reference signal (30.89 ppm).

Data from two-dimensional ^1^H, 13C HSQC experiments were acquired with a standard pulse program. Totals of 4,096 and 512 data points were collected in the F2 and F1 dimensions, respectively. An appropriate number of scans were accumulated prior to Fourier transformation to yield digital resolutions of 0.2 Hz and 1.0 Hz per point in F2 and F1, respectively.

NMR measurements shown in Fig. 3d and e, 4, and 5 were recorded on a Bruker Avance III HD 600 MHz spectrometer with a QXI room-temperature probe for ^1^H/^13^C/^15^N/^31^P (Bruker Biospin, Germany). Polymers were dissolved in 500 µl and measured in standard 5-mm TA tubes (Armar, Germany) at 298 K. The temperature was calibrated with methanol-d_4_ (Armar, Germany) (99.8% D), and spectra were calibrated in ^1^H using the Bruker standard sample of 2 mM sucrose–0.5 mM DSS (4,4-dimethyl-4-silapentane-1-sulfonic acid). ^13^C frequencies were calibrated indirectly using the recommended scaling factor Ξ of 0.25144953. Indirect referencing of 31P chemical shifts was performed using the chemical shift ratio 0.404808636 as advised by the Biological Magnetic Resonance Data Bank. ^1^H NMR spectra were recorded with 64,000 data points and a spectral width of 20 ppm, typically using 64 transients. ^31^P NMR spectra were recorded with 16,000 data points and a spectral width of 50 ppm, typically using 64 transients. 2D ^1^H-^13^C HSQC spectra were measured using the Bruker pulse sequence hsqcedetgpsisp2.2 with 2,048- and 16-ppm spectral widths for ^1^H and 230 data points and a 60-ppm spectral width using 32 scans and a recycle delay of 1.5 s, resulting in a measurement time of 3.5 h. 2D ^1^H-^1^H TOCSY spectra were collected with 2,-048- and 13.9-ppm spectral widths for ^1^H and 512 data points and a 13.9-ppm spectral width using 4 scans, a mixing time of 80 ms, and a recycle delay of 2 s, resulting in a measurement time of 1.5 h. 2D ^1^H-^1^H COSY spectra were collected using cosygpppqf with 2,048- and 10-ppm spectral widths for ^1^H and 128 data points and a 10-ppm spectral width using 32 scans and a recycle delay of 2 s, resulting in a measurement time of 2 h 40 min. 2D ^1^H-^31^P HMBC spectra were recorded using the pulse sequence hmbclpndqf with 4,096- and 10-ppm spectral widths for ^1^H and 64 data points and a 30.5-ppm spectral width using 32 scans and a recycle delay of 1.5 s, resulting in a measurement time of 1 h 12 min. 2D ^1^H-^1^H NOESY spectra were collected with 2,048- and 10-ppm spectral widths for ^1^H and 700 data points and a 10-ppm spectral width using 32 scans, a mixing time of 120 ms, and a recycle delay of 1 s, resulting in a measurement time of 8 h 20 min. 2D ^1^H-^13^C HMBC spectra were recorded using the pulse sequence hmbclpndqf with 4,096- and 20-ppm spectral widths for ^1^H and 512 data points and a 222-ppm spectral width using 64 scans, optimized for a J_CH_ long-range coupling of 8 Hz and a recycle delay of 2 s, resulting in a measurement time of 20.5 h.

Bruker TopSpin versions 3.5pl6 and 3.2 were used to process NMR data. Topspin and Sparky (T. D. Goddard and D. G. Kneller, SPARKY 3; University of California, San Francisco) were used to analyze and assign NMR data.

### Accession number(s).

Accession numbers KY798410 and KY807157 (see Table 2) have been submitted to GenBank. All other accession numbers cited in the manuscript are already accessible.
