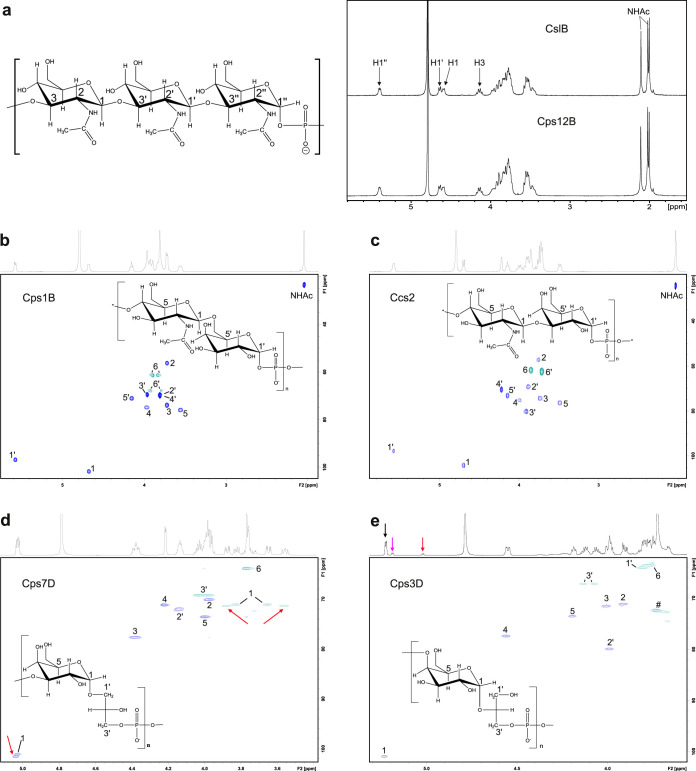# Erratum for Litschko et al., “A New Family of Capsule Polymerases Generates Teichoic Acid-Like Capsule Polymers in Gram-Negative Pathogens”

**DOI:** 10.1128/mBio.02433-20

**Published:** 2020-10-06

**Authors:** Christa Litschko, Davide Oldrini, Insa Budde, Monika Berger, Jochen Meens, Rita Gerardy-Schahn, Francesco Berti, Mario Schubert, Timm Fiebig

**Affiliations:** a Institute of Clinical Biochemistry, Hannover Medical School, Hannover, Germany; b GSK, Siena, Italy; c Institute for Microbiology, University of Veterinary Medicine Hannover, Hannover, Germany; d Department of Biosciences, University of Salzburg, Salzburg, Austria

## ERRATUM

Volume 9, no. 3, e00641-18, 2018, https://doi.org/10.1128/mBio.00641-18. In Fig. 1b, the polymer structures of Actinobacillus pleuropneumoniae serotypes 3 and 7 were swapped. In Fig. 2c, the linkage between GlcNAc and Gal in the polymer structure shown in the chromatogram was incorrectly written as “β3” and should be “β6.” In Fig. 3e, the polymer structure showed a linkage between phosphate and O3 of galactose but should show a linkage between phosphate and O4 of galactose. These changes do not change the conclusions of the paper. The corrected figures appear below. We thank Julia Schulze (Hannover Medical School, Germany) and Olga G. Ovchinnikova and Chris Whitfield (both University of Guelph, Canada) for making us aware of these mistakes.

**FIG 1:**
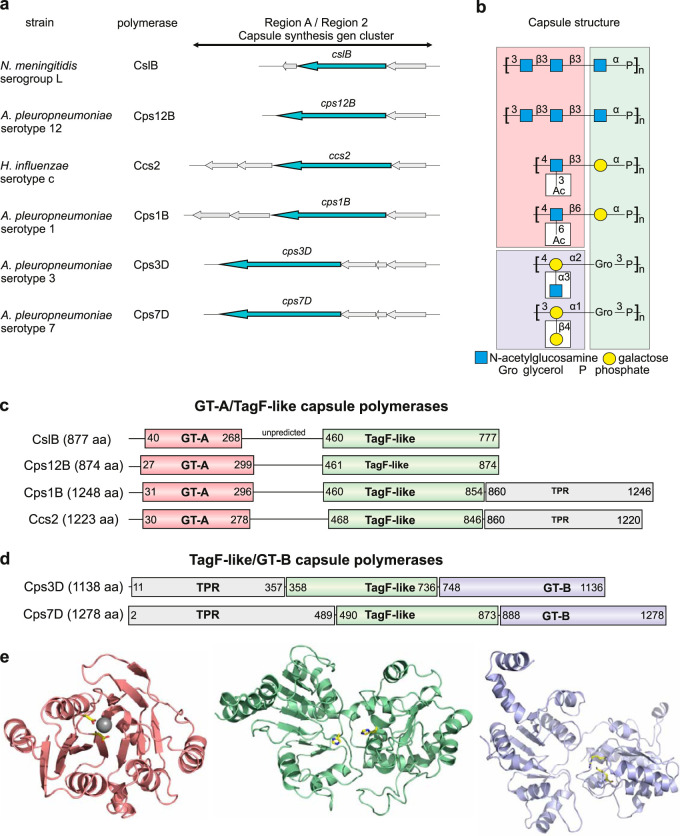


**FIG 2:**
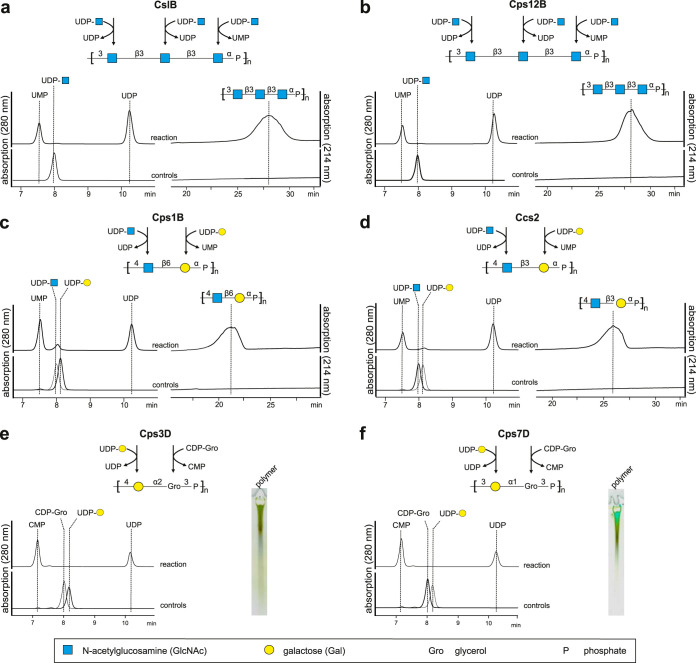


**FIG 3:**