# Supplementary material for: A New Family of Capsule Polymerases Generates Teichoic Acid-Like Capsule Polymers in Gram-Negative Pathogens
Source: mBio. 2018 May 29;9(3):e00641-18. doi: 10.1128/mBio.00641-18 (PMC5974469; doi:10.1128/mBio.00641-18)
Supplement: FIG S1 [file mbo003183904sf1.pdf]

Fig. S1

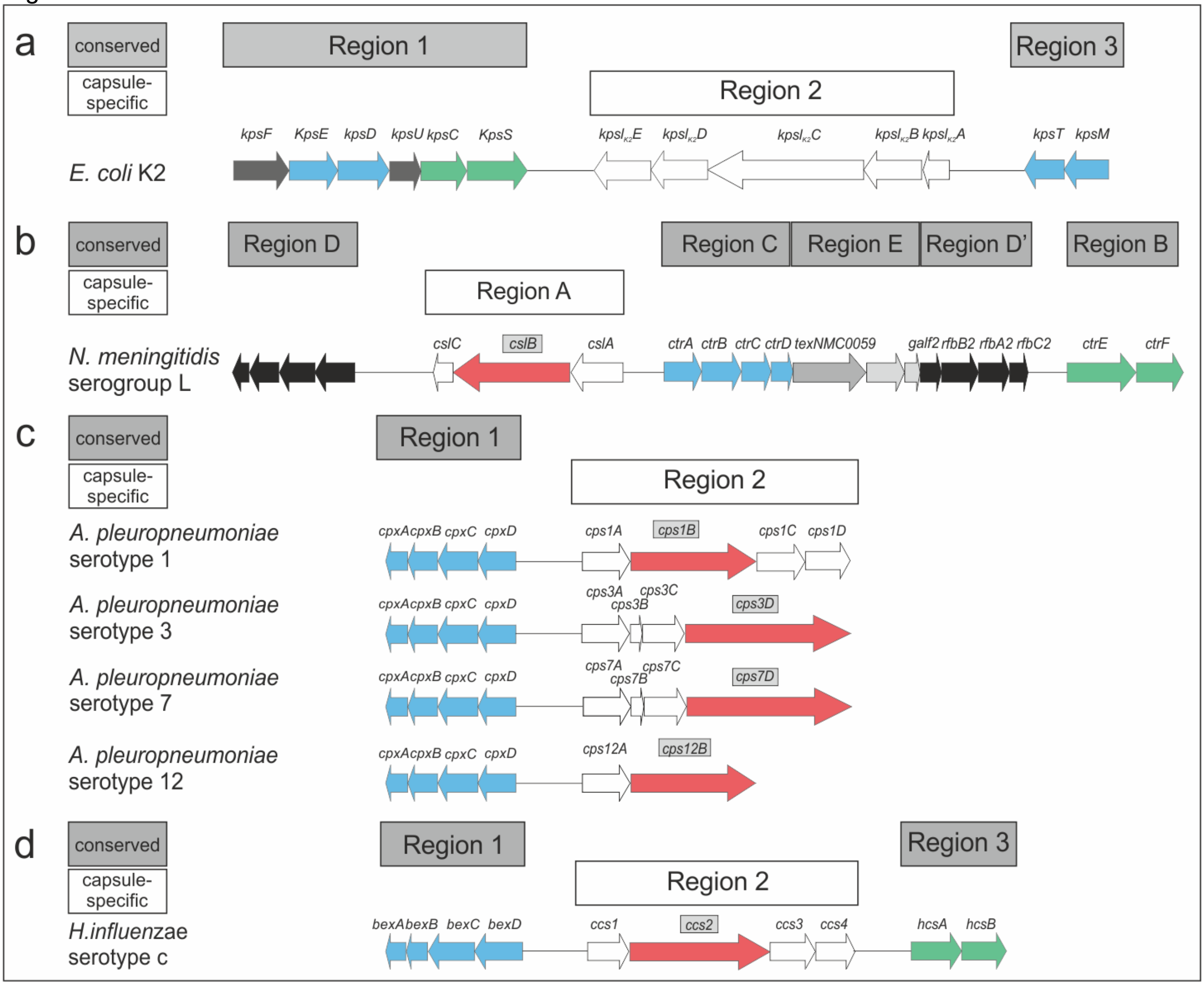

Fig. S1: Schematic overview of the capsule gene clusters of **a** *E. coli* K2 (modified from E. L. Buckles, X. Wang, M. C. Lane, C. V. Lockatell, D. E. Johnson, D. A. Rasko, H. L. T. Mobley, M. S. Donnenberg, J Infect Dis 199:1689–97, 2009), **b** *N. meningitidis* serogroup L (modified from O. B. Harrison, H. Claus, Y. Jiang, J. S. Bennett, H. B. Bratcher, K. A. Jolley, C. Corton, R. Care, J. T. Poolman, W. D. Zollinger, et al., Emerg Infect Dis 19:566–73, 2013), **c** *A. pleuropneumoniae* serotypes 1, 3, 7 and 12 (modified from H. Ito, J Vet Med Sci 77:583–6, 2015; S. G. Jessing, P. Ahrens, T. J. Inzana, Ø. Angen, Vet Microbiol 129:350–9, 2008; Z. Xu, X. Chen, L. Li, T. Li, S. Wang, H. Chen, R. Zhou, J Bacteriol 192:5625–36, 2010) and **d** *H. influenzae* serotype c (modified from S. W. Satola, P. L. Schirmer, M. M. Farley, Infect Immun 71:3639–3644, 2003; T.-T. Lâm, H. Claus, M. Frosch, U. Vogel, Res Microbiol 162:483–7, 2011; S. Sukupolvi-Petty, S. Grass, J. W. St Geme, III, J Bacteriol 188:3870–7, 2006). The gene clusters are divided into conserved regions (grey boxes) and capsule-specific regions (white boxes). The conserved regions encode proteins necessary for translocation (green) and export (blue) of the capsule polymer to the cell surface. Genes encoding capsule polymerases are highlighted in red and localized in the capsule specific region. Genes and interspaces in this scheme are not drawn to scale. Graphical representation in the style of B. F. Cress, J. A. Englaender, W. He, D. Kasper, R. J. Linhardt, M. A. G. Koffas, FEMS Microbiol Rev 38:660–697, 2014.
