# Supplementary material for: A New Family of Capsule Polymerases Generates Teichoic Acid-Like Capsule Polymers in Gram-Negative Pathogens
Source: mBio. 2018 May 29;9(3):e00641-18. doi: 10.1128/mBio.00641-18 (PMC5974469; doi:10.1128/mBio.00641-18)
Supplement: FIG S2 [file mbo003183904sf2.pdf]

Fig. S2

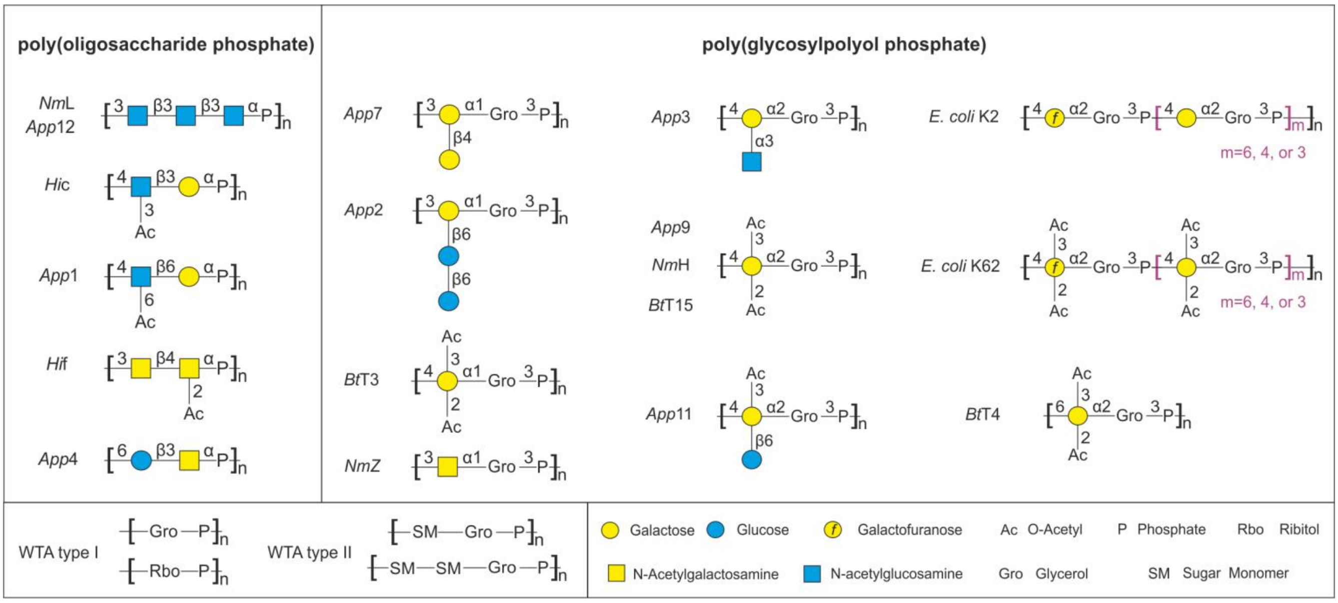

Fig. S2: Capsule structures of group 2 capsule expressing bacteria that encode TagF-like polymerases. Branching mono- and oligosaccharides as well as O-acetyl groups are usually introduced by separate enzymes. Schematics of wall teichoic acid (WTA) type I and II are depicted for comparison and displayed according to I. B. Naumova, A. S. Shashkov, E. M. Tul'skaya, G. M. Streshinskaya, Y. I. Kozlova, N. V Potekhina, L. I. Evtushenko, E. Stackebrandt, *FEMS Microbiol Rev 25:*269–84, 2001. To allow a concise display, bacterial species are abbreviated in italics and serogroup/serotype classification is added in regular font. Abbreviations used are: *App*, *Actinobacillus pleuropneumoniae*; *Bt*, *Bibersteinia trehalosi*; *Hi*, *Haemophilus influenza*; *Nm*, *Neisseria meningitidis*.
