# Supplementary material for: A New Family of Capsule Polymerases Generates Teichoic Acid-Like Capsule Polymers in Gram-Negative Pathogens
Source: mBio. 2018 May 29;9(3):e00641-18. doi: 10.1128/mBio.00641-18 (PMC5974469; doi:10.1128/mBio.00641-18)
Supplement: FIG S3 [file mbo003183904sf3.pdf]

Fig. S3

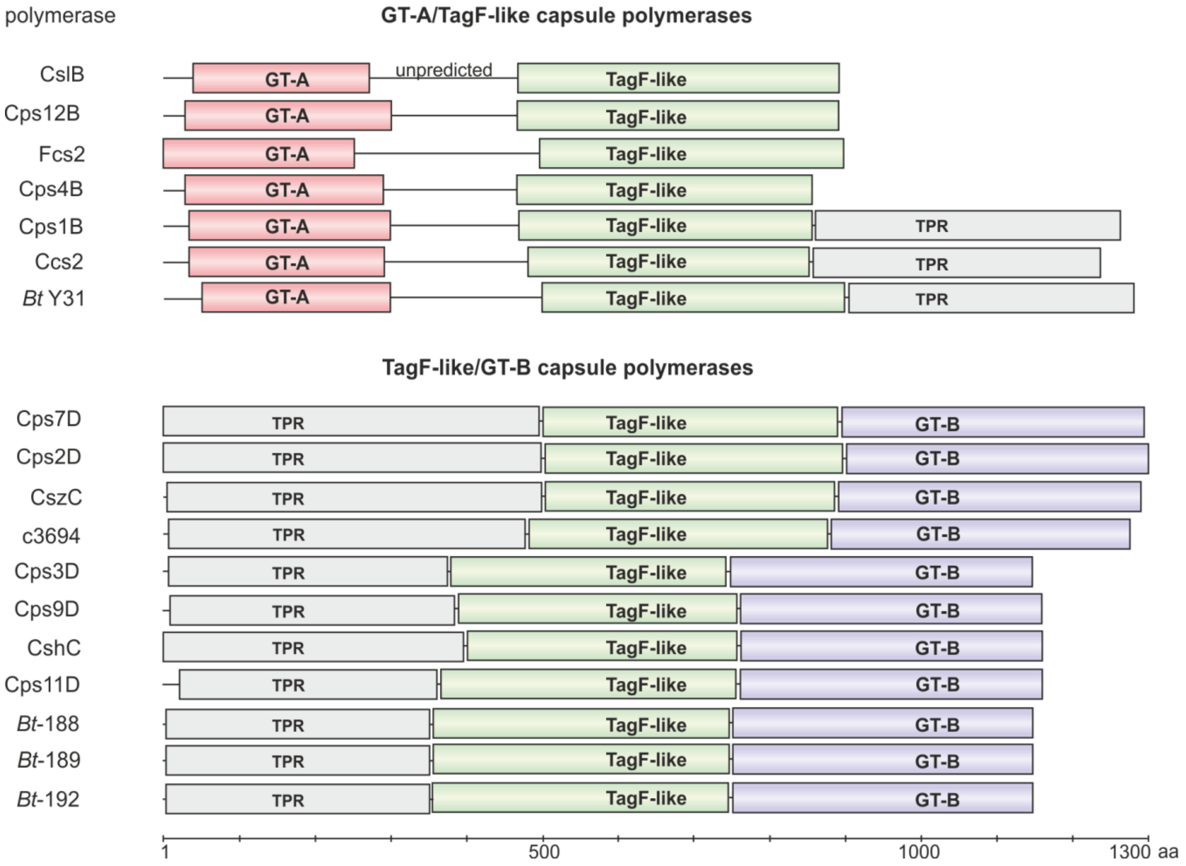

Fig. S3: Overview of the predicted architecture of all TagF-like polymerases analyzed in this study. Homology modeling was performed using the structure prediction tool PHYRE2 (L. A. Kelley, S. Mezulis, C. M. Yates, M. N. Wass, M. J. E. Sternberg, Nat Protoc 10: 845–58, 2015.). The name of the polymerase is displayed in front of each model. A ruler (bottom) indicates the length of each polypeptide as well as the sequence coverage of each modelled domain. The following protein sequences were submitted to PHYRE2: CslB of *N. meningitidis* serogroup L (uniprot: Q9RGQ9), Cps1B of *A. pleuropneumoniae* serotype 1 (uniprot: E0EA77), Cps12B of *A. pleuropneumoniae* serotype 12 (uniprot: Q69AA8), Ccs2 of *H. influencae* serotype c (GenBank: AEC50903.1), Fcs2 of *H. influencae* serotype f (GenBank: AAQ12660.1), Cps4B of *A. pleuropneumoniae* serotype 4 (uniprot: F4YBG0), *Bt* Y31 of the non-serotyped *Bibersteinia trehalosi* strain Y31 (GenBank: OAQ14264.1), Cps7D of *A. pleuropneumoniae* serotype 7 (GenBank: ACE62291.1), Cps2D of *A. pleuropneumoniae* serotype 2 (uniprot: Q6UYC4), CszC of *N. meningitidis* serogroup Z (uniprot: Q5QRV6), Cps3D of *A. pleuropneumoniae* serotype 3 (GenBank: KY807157), Cps9D of *A. pleuropneumoniae* serotype 9 (uniprot: E0F019), CshC of *N. meningitidis* serogroup H (uniprot: H6T5X6), Cps11D of *A. pleuropneumoniae* serotype 11 (uniprot: E0FCQ3), *Bt*-188 of the non-serotyped *Bibersteinia trehalosi* strain USDA-ARS-USMARC-188 (GenBank: AHG82487.1), *Bt*-189 of the non-serotyped *Bibersteinia trehalosi* strain USDA-ARS-USMARC-189 (GenBank: AHG84818.1), *Bt*-192 of the non-serotyped *Bibersteinia trehalosi* strain USDA-ARS-USMARC-192 (GenBank: AGH37704.1) and c3694 of *E. coli* K2 strain CFT073 (GenBank: AAN82142.1).
