# Supplementary material for: A New Family of Capsule Polymerases Generates Teichoic Acid-Like Capsule Polymers in Gram-Negative Pathogens
Source: mBio. 2018 May 29;9(3):e00641-18. doi: 10.1128/mBio.00641-18 (PMC5974469; doi:10.1128/mBio.00641-18)
Supplement: FIG S4 [file mbo003183904sf4.pdf]

Fig. S4

	TagF	CshC	Bt189	Bt188	Bt192	Cps3D	Cps9D	Cps11D	c3694	CszC	Cps7D	Cps2D	Fcs2	Cps1B	BtY31	Ccs2	Cps4B	CslB	Cps12B
TagF	100.00	28.57	27.96	28.46	28.15	27.64	28.30	28.61	29.14	28.93	27.73	28.53	16.62	18.10	19.51	17.59	18.60	18.45	17.42
CshC	28.57	100.00	68.32	68.78	67.89	71.16	70.87	70.57	47.23	44.29	41.58	44.77	16.06	18.77	17.13	17.90	17.68	15.52	17.72
Bt189	27.96	68.32	100.00	98.67	97.91	77.45	77.37	77.23	48.54	44.81	44.83	46.90	17.68	21.98	17.85	19.57	18.40	14.71	17.22
Bt188	28.46	68.78	98.67	100.00	99.21	77.45	77.87	77.78	48.40	45.08	44.71	46.36	18.04	21.98	18.15	19.88	18.71	15.32	17.18
Bt192	28.15	67.89	97.91	99.21	100.00	77.45	77.37	77.28	47.75	45.08	44.71	46.36	17.99	21.98	17.85	19.57	18.40	15.02	17.22
Cps3D	27.64	71.16	77.45	77.45	77.45	100.00	92.29	92.06	44.27	44.14	42.86	44.35	17.07	20.99	16.87	18.27	16.51	14.67	18.65
Cps9D	28.30	70.87	77.37	77.87	77.37	92.29	100.00	100.00	44.83	43.05	42.55	43.82	17.33	20.99	16.87	18.89	16.82	15.27	18.67
Cps11D	28.61	70.57	77.23	77.78	77.28	92.06	100.00	100.00	44.71	43.05	42.22	43.82	17.33	20.99	16.87	18.89	16.82	15.27	18.67
c3694	29.14	47.23	48.54	48.40	47.75	44.27	44.83	44.71	100.00	44.99	48.55	48.40	17.82	19.63	17.38	20.00	17.33	18.99	18.92
CszC	28.93	44.29	44.81	45.08	45.08	44.14	43.05	43.05	44.99	100.00	69.89	70.62	16.27	17.99	16.67	18.35	16.92	16.52	17.47
Cps7D	27.73	41.58	44.83	44.71	44.71	42.86	42.55	42.22	48.55	69.89	100.00	87.53	17.77	18.60	16.36	17.13	20.24	17.99	18.67
Cps2D	28.53	44.77	46.90	46.36	46.36	44.35	43.82	43.82	48.40	70.62	87.53	100.00	16.92	19.82	17.88	19.27	19.94	17.99	19.34
Fcs2	16.62	16.06	17.68	18.04	17.99	17.07	17.33	17.33	17.82	16.27	17.77	16.92	100.00	30.13	26.91	30.58	26.77	30.18	32.55
Cps1B	18.10	18.77	21.98	21.98	21.98	20.99	20.99	20.99	19.63	17.99	18.60	19.82	30.13	100.00	51.91	50.66	50.13	31.17	31.61
BtY31	19.51	17.13	17.85	18.15	17.85	16.87	16.87	16.87	17.38	16.67	16.36	17.88	26.91	51.91	100.00	50.53	54.43	31.12	31.63
Ccs2	17.59	17.90	19.57	19.88	19.57	18.27	18.89	18.89	20.00	18.35	17.13	19.27	30.58	50.66	50.53	100.00	52.91	30.67	32.71
Cps4B	18.60	17.68	18.40	18.71	18.40	16.51	16.82	16.82	17.33	16.92	20.24	19.94	26.77	50.13	54.43	52.91	100.00	30.36	29.74
CslB	18.45	15.52	14.71	15.32	15.02	14.67	15.27	15.27	18.99	16.52	17.99	17.99	30.18	31.17	31.12	30.67	30.36	100.00	51.36
Cps12B	17.42	17.72	17.22	17.18	17.22	18.65	18.67	18.67	18.92	17.47	18.67	19.34	32.55	31.61	31.63	32.71	29.74	51.36	100.00

Fig. S4: Sequence identity matrix (in %) based on a Clustal Omega multiple sequence alignment of all predicted TagF-like domains (as they are shown in Fig. S3) and the modelling template TagF.
