# Supplementary material for: A New Family of Capsule Polymerases Generates Teichoic Acid-Like Capsule Polymers in Gram-Negative Pathogens
Source: mBio. 2018 May 29;9(3):e00641-18. doi: 10.1128/mBio.00641-18 (PMC5974469; doi:10.1128/mBio.00641-18)
Supplement: FIG S5 [file mbo003183904sf5.pdf]

Fig. S5

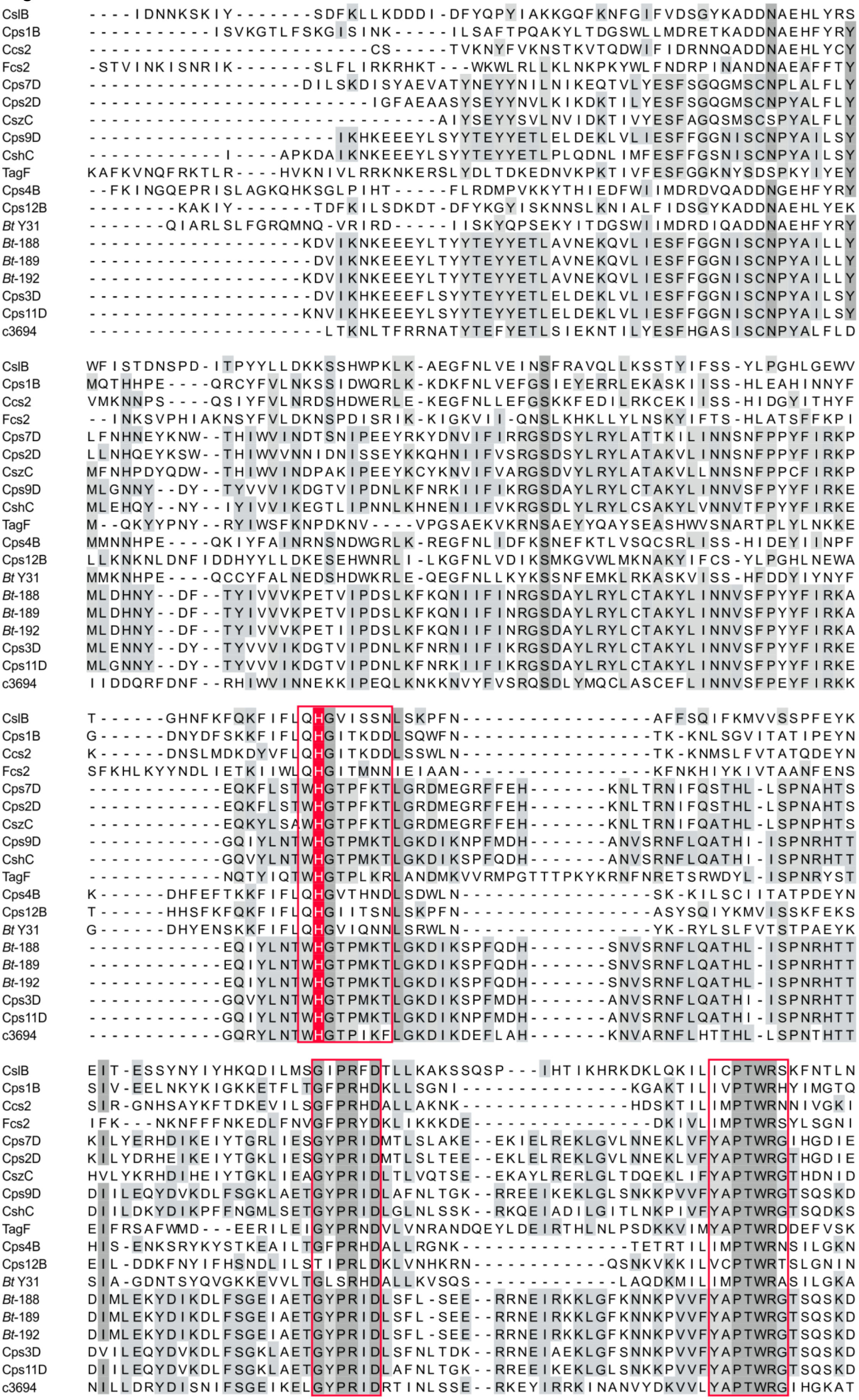
CslB ----IDNNKSKIY-------SDFKLLKDDDI-DFYQPYIAKKGQFKNFGIFVDSGYKADDNAEHLYRS
Cps1B -----------ISVKGTLFSKGISINK-----ILSAFTPQAKYLTDGSWLLMDRETKADDNAEHFYRY
Ccs2 -------------------------CS-----TVKNYFVKNSTKVTQDWIFIDRNNQADDNAEHLYCY
Fcs2 -STVINKISNRIK-------SLFLIRKRHKT---WKWLRLLKLNKPKYWLFNDRPINANDNAEAFFTY
Cps7D ------------------DILSKDISYAEVATYNEYYNILNIKEQTVLYESFSGQGMSCNPLALFLY
Cps2D --------------------------IGFAEAASYSEYYNVLKIKDKTILYESFSGQGMSCNPYALFLY
CszC -----------------------------AIYSEYYSVLNVIDKTIVYESFAGQSMSCSPYALFLY
Cps9D ----------------------IKHKEEEYLSYYTEYYETLELDEKLVLIESFFGGNISCNPYAILSY
CshC ------------I----APKDAIKNKEEEYLSYYTEYYETLPLQDNLIMFESFFGSNISCNPYAILSY
TagF KAFKVNQFRKTLR----HVKNIVLRRKNKERSLYDLTDKEDNVKPKTIVFESFGGKNYSDSPKYIYEY
Cps4B --FKINGQEPRISLAGKQHKSGLPIHT-----FLRDMPVKKYTHIEDFWIIMDRDVQADDNGEHFYRY
Cps12B --------KAKIY-------TDFKILSDKDT-DFYKGYISKNNSLKNIALFIDSGYKADDNAEHLYEK
Bt Y31 -------QIARLSLFGRQMNQ-VRIRD-----IISKYQPSEKYITDGSWIIMDRDIQADDNAEHFYRY
Bt-188 ------------------KDVIKNKEEEYLTYYTEYYETLAVNEKQVLIESFFGGNISCNPYAILLY
Bt-189 -------------------DVIKNKEEEYLTYYTEYYETLAVNEKQVLIESFFGGNISCNPYAILLY
Bt-192 ------------------KDVIKNKEEEYLTYYTEYYETLAVNEKQVLIESFFGGNISCNPYAILLY
Cps3D -------------------DVIKHKEEEYLSYYTEYYETLELDEKLVLIESFFGGNISCNPYAILSY
Cps11D ------------------KNVIKHKEEEYLSYYTEYYETLELDEKLVLIESFFGGNISCNPYAILSY
c3694 ---------------------LTKNLTFRRNATYTEFYETLSIEKNTILYESFHGASISCNPYALFLD

CslB WFISTDNSPD-ITPYYLLDKKSSHWPKLK-AEGFNLVEINSFRAVQLLKSSTYIFSS-YLPGHLGEWV
Cps1B MQTHHPE----QRCYFVLNKSSIDWQRLK-KDKFNLVEFGSIEYERRLEKASKIISS-HLEAHINNYF
Ccs2 VMKNNPS----QSIYFVLNRDSHDWERLE-KEGFNLLEFGSKKFEDILRKCEKIISS-HIDGYITHYF
Fcs2 --INKSVPHIAKNSYFVLDKNSPDISRIK-KIGKVII-QNSLKHKLLYLNSKYIFTS-HLATSFFKPI
Cps7D LFNHNEYKNW--THIWVINDTSNIPEEYRKYDNVIFIRRGSDSYLRYLATTKILINNSNFPPYFIRKP
Cps2D LLNHQEYKSW--THIWVVNNIDNISSEYKKQHNIIFVSRGSDSYLRYLATAKVLINNSNFPPYFIRKP
CszC MFNHPDYQDW--THIWVINDPAKIPEEYKCYKNVIFVARGSDVYLRYLATAKVLLNNSNFPPCFIRKP
Cps9D MLGNNY--DY--TYVVVIKDGTVIPDNLKFNRKIIFIKRGSDAYLRYLCTAKYLINNVSFPYYFIRKE
CshC MLEHQY--NY--IYIVVIKEGTLIPNNLKHNENIIFVKRGSDLYLRYLCSAKYLVNNVTFPYYFIRKE
TagF M--QKYYPNY--RYIWSFKNPDKNV----VPGSAEKVKRNSAEYYQAYSEASHWVSNARTPLYLNKKE
Cps4B MMNNHPE----QKIYFAINRNSNDWGRLK-REGFNLIDFKSNEFKTLVSQCSRLISS-HIDEYIINPF
Cps12B LLKNKNLDNFIDDHYYLLDKESEHWNRLI-LKGFNLVDIKSMKGVWLMKNAKYIFCS-YLPGHLNEWA
Bt Y31 MMKNHPE----QCCYFALNEDSHDWKRLE-QEGFNLLKYKSSNFEMKLRKASKVISS-HFDDYIYNYF
Bt-188 MLDHNY--DF--TYIVVVKPETVIPDSLKFKQNIIFINRGSDAYLRYLCTAKYLINNVSFPYYFIRKA
Bt-189 MLDHNY--DF--TYIVVVKPETVIPDSLKFKQNIIFINRGSDAYLRYLCTAKYLINNVSFPYYFIRKA
Bt-192 MLDHNY--DF--TYIVVVKPETIIPDSLKFKQNIIFINRGSDAYLRYLCTAKYLINNVSFPYYFIRKA
Cps3D MLENNY--DY--TYVVVIKDGTVIPDNLKFNRNIIFIKRGSDAYLRYLCTAKYLINNVSFPYYFIRKE
Cps11D MLGNNY--DY--TYVVVIKDGTVIPDNLKFNRKIIFIKRGSDAYLRYLCTAKYLINNVSFPYYFIRKE
c3694 IIDDQRFDNF--RHIWVINNEKKIPEQLKNKKNVYFVSRQSDLYMQCLASCEFLINNVSFPEYFIRKK

CslB T------GHNFKFQKFIFLQHGVISSNLSKPFN----------------AFFSQIFKMVVSSPFEYK
Cps1B G------DNYDFSKKFIFLQHGITKDDLSQWFN----------------TK-KNLSGVITATIPEYN
Ccs2 K------DNSLMDKDYVFLQHGITKDDLSSWLN----------------TK-KNMSLFVTATQDEYN
Fcs2 SFKHLKYYNDLIETKIIWLQHGITMNNIEIAAN----------------KFNKHIYKIVTAANFENS
Cps7D ------------EQKFLSTWHGTPFKTLGRDMEGRFFEH-------KNLTRNIFQSTHL-LSPNAHTS
Cps2D ------------EQKFLSTWHGTPFKTLGRDMEGRFFEH-------KNLTRNIFQSTHL-LSPNAHTS
CszC ------------EQKYLSAWHGTPFKTLGRDMEGRFFEH-------KNLTRNIFQATHL-LSPNPHTS
Cps9D ------------GQIYLNTWHGTPMKTLGKDIKNPFMDH-------ANVSRNFLQATHI-ISPNRHTT
CshC ------------GQVYLNTWHGTPMKTLGKDIKSPFQDH-------ANVSRNFLQATHI-ISPNRHTT
TagF ------------NQTYIQTWHGTPLKRLANDMKVVRMPGTTTPKYKRNFNRETSRWDYL-ISPNRYST
Cps4B K------DHFEFTKKFIFLQHGVTHNDLSDWLN----------------SK-KILSCIITATPDEYN
Cps12B T------HHSFKFQKFIFLQHGIITSNLSKPFN----------------ASYSQIYKMVISSKFEKS
Bt Y31 G------DHYENSKKFIFLQHGVIQNNLSRWLN----------------YK-RYLSLFVTSTPAEYK
Bt-188 ------------EQIYLNTWHGTPMKTLGKDIKSPFQDH-------SNVSRNFLQATHL-ISPNRHTT
Bt-189 ------------EQIYLNTWHGTPMKTLGKDIKSPFQDH-------SNVSRNFLQATHL-ISPNRHTT
Bt-192 ------------EQIYLNTWHGTPMKTLGKDIKSPFQDH-------SNVSRNFLQATHL-ISPNRHTT
Cps3D ------------GQVYLNTWHGTPMKTLGKDIKSPFMDH-------ANVSRNFLQATHI-ISPNRHTT
Cps11D ------------GQIYLNTWHGTPMKTLGKDIKNPFMDH-------ANVSRNFLQATHI-ISPNRHTT
c3694 ------------GQRYLNTWHGTPIKFLGKDIKDEFLAH-------KNVARNFLHTTHL-LSPNTHTT

CslB EIT-ESSYNYIYHKQDILMSGIPRFDTLLKAKSSQSP---IHTIKHRKDKLQKILICPTWRSKFNTLN
Cps1B SIV-EELNKYKIGKKETFLTGFPRHDKLLSGNI--------------KGAKTILIVPTWRHYIMGTQ
Ccs2 SIR-GNHSAYKFTDKEVILSGFPRHDALLAKNK--------------HDSKTILIMPTWRNNIVGKI
Fcs2 IFK---NKNFFFNKEDLFNVGFPRYDKLIKKKDE--------------DKIVLIMPTWRSYLSGNI
Cps7D KILYERHDIKEIYTGRLIESGYPRIDMTLSLAKE--EKIELREKLGVLNNEKLVFYAPTWRGIHGDIE
Cps2D KILYDRHEIKEIYTGKLIESGYPRIDMTLSLTEE--EKLELREKLGVLNNEKLVFYAPTWRGTHGDIE
CszC HVLYKRHDIHEIYTGKLIEAGYPRIDLTLVQTSE--EKAYLRERLGLTDQEKLIFYAPTWRGTHDNID
Cps9D DIILEQYDVKDLFSGKLAETGYPRIDLAFNLTGK--RREEIKEKLGLSNKKPVVFYAPTWRGTSQSKD
CshC DIILDKYDIKPFFNGMLSETGYPRIDLGLNLSSK--RKQEIADILGITLNKPIVFYAPTWRGTSQDKS
TagF EIFRSAFWMD---EERILEIGYPRNDVLVNRANDQEYLDEIRTHLNLPSDKKVIMYAPTWRDDEFVSK
Cps4B HIS-ENKSRYKYSTKEAILTGFPRHDALLRGNK--------------TETRTILIMPTWRNSILGKN
Cps12B EIL-DDKFNYIFHSNDLILSTIPRLDKLVNHKRN------------QSNKVKKILVCPTWRTSLGNIN
Bt Y31 SIA-GDNTSYQVGKKEVVLTGLSRHDALLKVSQS-------------LAQDKMILIMPTWRASILGKA
Bt-188 DIMLEKYDIKDLFSGEIAETGYPRIDLSFL-SEE--RRNEIRKKLGFKNNKPVVFYAPTWRGTSQSKD
Bt-189 DIMLEKYDIKDLFSGEIAETGYPRIDLSFL-SEE--RRNEIRKKLGFKNNKPVVFYAPTWRGTSQSKD
Bt-192 DIMLEKYDIKDLFSGEIAETGYPRIDLSFL-SEE--RRNEIRKKLGFKNNKPVVFYAPTWRGTSQSKD
Cps3D DVILEQYDVKDLFSGKLAETGYPRIDLSFNLTDK--RRNEIAEKLGFSNNKPVVFYAPTWRGTSQSKD
Cps11D DIILEQYDVKDLFSGKLAETGYPRIDLAFNLTGK--RREEIKEKLGLSNKKPVVFYAPTWRGTSQSKD
c3694 NILLDRYDISNIFSGEIKELGYPRIDRTINLSSE--RKEYIRRKINANVYDKVVLYAPTWRGIHGKAT

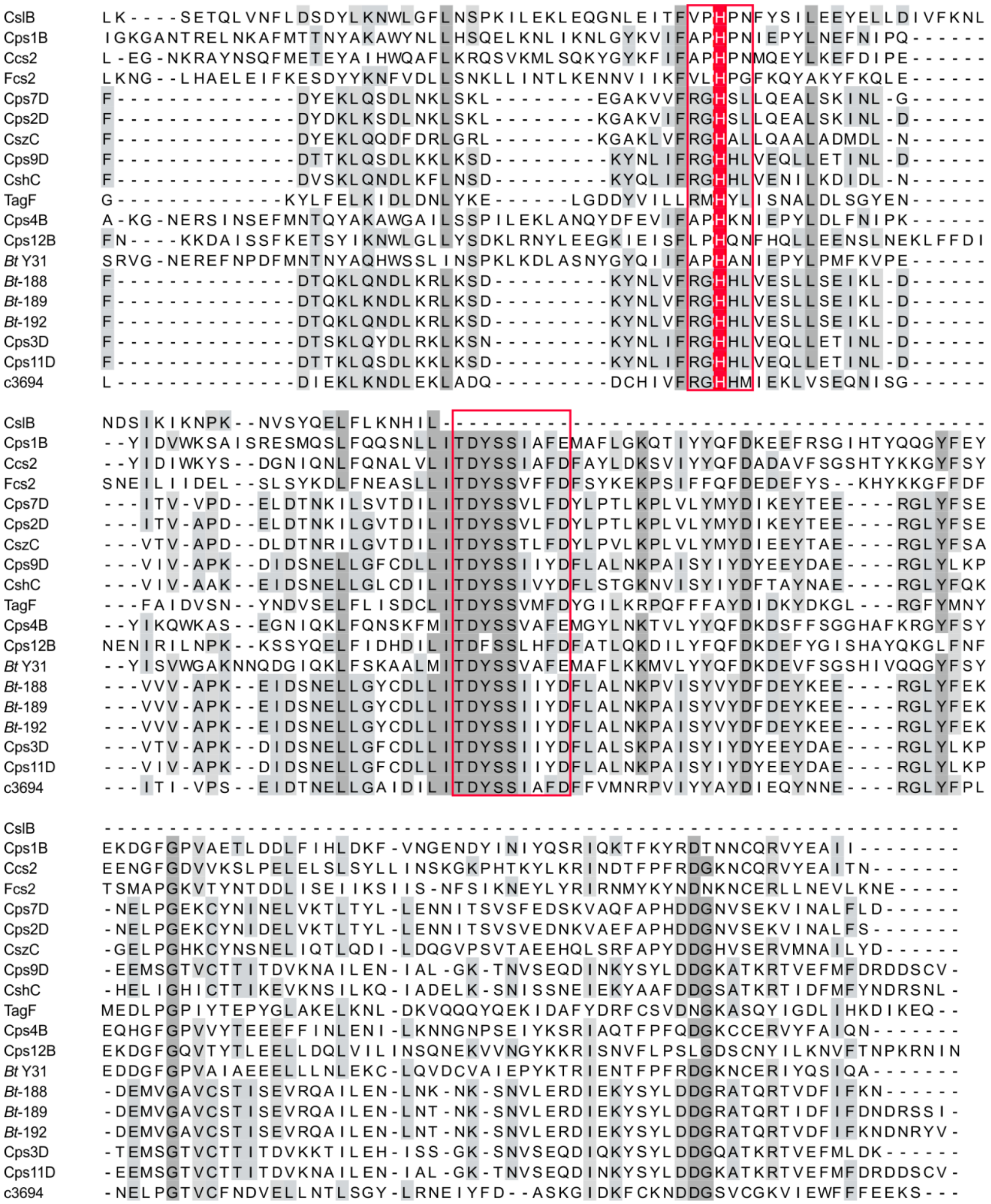

Fig. S5: Sequence alignment of all predicted TagF-like domains analyzed in this study including the sequence of the template TagF of *Staphylococcus epidermidis* (uniprot: Q5HLM5) used for PHYRE2 modeling. Database references for all TagF-like polymerase sequences are indicated in the figure legend of Supplementary Figure 3. Identical amino acids are shown in grey boxes and the conserved histidine residues are highlighted in red. The conserved active site motifs reported for TagF are shown in boxed sections (red). The sequence alignment was performed with Clustal Omega (F. Sievers, A. Wilm, D. Dineen, T. J. Gibson, K. Karplus, W. Li, R. Lopez, H. McWilliam, M. Remmert, J. Söding, J. D. Thompson, D. G. Higgins, Mol Syst Biol 7:539, 2011) on the uniprot website (http://www.uniprot.org/align/) (E. Boutet, D. Lieberherr, M. Tognolli, M. Schneider, A. Bairoch, Methods Mol Biol 406:89–112, 2007) and annotated with the Jalview software (A. M. Waterhouse, J. B. Procter, D. M. A. Martin, M. Clamp, G. J. Barton, Bioinformatics 25:1189–91, 2009).
