# Supplementary material for: A New Family of Capsule Polymerases Generates Teichoic Acid-Like Capsule Polymers in Gram-Negative Pathogens
Source: mBio. 2018 May 29;9(3):e00641-18. doi: 10.1128/mBio.00641-18 (PMC5974469; doi:10.1128/mBio.00641-18)
Supplement: FIG S6 [file mbo003183904sf6.pdf]

Fig. S6

a

	K4CP	Fcs2	CslB	Cps12B	BtY31	Cps1B	Ccs2	Cps4B
K4CP	100.00	19.55	18.84	20.82	20.38	17.96	17.47	18.10
Fcs2	19.55	100.00	32.09	32.22	27.31	31.25	30.04	30.38
CslB	18.84	32.09	100.00	69.47	41.59	47.11	45.29	46.22
Cps12B	20.82	32.22	69.47	100.00	42.55	44.87	38.96	41.57
BtY31	20.38	27.31	41.59	42.55	100.00	54.11	47.37	49.79
Cps1B	17.96	31.25	47.11	44.87	54.11	100.00	54.84	54.37
Ccs2	17.47	30.04	45.29	38.96	47.37	54.84	100.00	58.63
Cps4B	18.10	30.38	46.22	41.57	49.79	54.37	58.63	100.00

b

	TarM	CshC	Cps3D	Cps9D	Cps11D	Bt189	Bt188	Bt192	c3694	CszC	Cps7D	Cps2D
TarM	100.00	22.04	23.03	23.19	23.10	27.05	25.76	25.99	21.95	22.02	22.66	21.93
CshC	22.04	100.00	70.26	70.66	70.95	70.69	64.62	65.63	46.09	40.95	41.28	40.33
Cps3D	23.03	70.26	100.00	92.03	92.03	75.84	68.89	69.51	45.29	40.87	41.49	41.49
Cps9D	23.19	70.66	92.03	100.00	100.00	77.12	68.89	69.51	45.31	40.82	42.05	41.43
Cps11D	23.10	70.95	92.03	100.00	100.00	77.12	69.07	69.51	45.29	40.87	41.75	41.49
Bt189	27.05	70.69	75.84	77.12	77.12	100.00	80.15	79.84	46.60	41.65	40.98	41.49
Bt188	25.76	64.62	68.89	68.89	69.07	80.15	100.00	98.71	46.34	39.85	40.72	40.72
Bt192	25.99	65.63	69.51	69.51	69.51	79.84	98.71	100.00	46.46	40.57	41.09	40.83
c3694	21.95	46.09	45.29	45.31	45.29	46.60	46.34	46.46	100.00	46.89	49.09	49.09
CszC	22.02	40.95	40.87	40.82	40.87	41.65	39.85	40.57	46.89	100.00	72.12	67.65
Cps7D	22.66	41.28	41.49	42.05	41.75	40.98	40.72	41.09	49.09	72.12	100.00	83.12
Cps2D	21.93	40.33	41.49	41.43	41.49	41.49	40.72	40.83	49.09	67.65	83.12	100.00

Fig. S6: Sequence identity matrices (in %) based on a Clustal Omega multiple sequence alignment of **a** all predicted GT-A domains (as they are shown in Fig. S3) and the C-terminal domain of the modelling template K4CP (*E. coli* K4 polymerase) and **b** all predicted GT-B domains (as they are shown in Fig. S3) and the modelling template TarM.
