# Supplementary material for: A New Family of Capsule Polymerases Generates Teichoic Acid-Like Capsule Polymers in Gram-Negative Pathogens
Source: mBio. 2018 May 29;9(3):e00641-18. doi: 10.1128/mBio.00641-18 (PMC5974469; doi:10.1128/mBio.00641-18)
Supplement: FIG S7 [file mbo003183904sf7.pdf]

Fig. S7

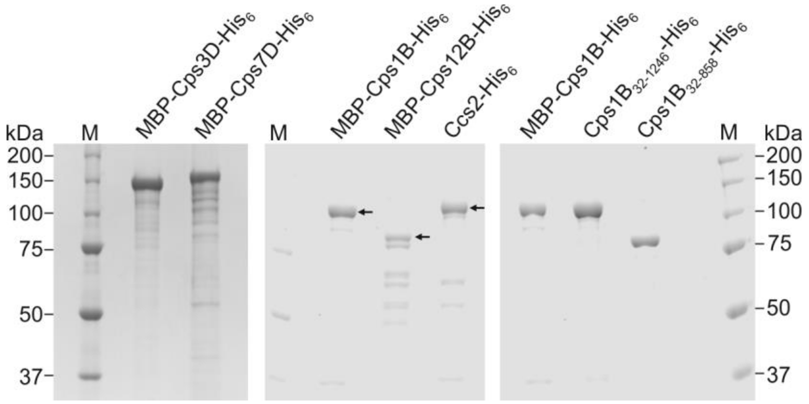

Fig. S7: Coomassie-stained SDS-polyacrylamide gel of TagF-like polymerases purified by affinity chromatography via their C-terminal His_6-tag. N-terminal fusion to maltose binding protein is indicated with ‘MBP’ in the construct name. 1.5-3 µg of protein were loaded per lane. MBP-Cps3D-His_6 (177 kDa) and MBP-Cps7D-His_6 (192 kDa) could be enriched as full-length constructs. Western blot analysis with an α-MBP antibody (data not shown) demonstrated that MBP-Cps1B-His_6 (190 kDa), MBP-Cps12B-His_6 (146 kDa) and Ccs2-His_6 (144 kDa) were exclusively purified as N-terminal degradation products (indicated by arrows), lacking the MBP-tag. N-terminal degradation is common for group 2 polymerases and usually does not interfere with activity (T. Fiebig, F. Freiberger, V. Pinto, M. R. Romano, A. Black, C. Litschko, A. Bethe, D. Yashunsky, R. Adamo, A. Nikolaev, F. Berti, R. Gerardy-Schahn, *J Biol Chem 289:*19395–407, 2014; C. Litschko, M. R. Romano, V. Pinto, H. Claus, U. Vogel, F. Berti, R. Gerardy-Schahn, T. Fiebig, *J Biol Chem 290:*24355–66, 2015). The dominant protein band purified from the MBP-Cps1B-His_6 expression culture was N-terminally sequenced and could be identified as ΔN31 truncation of Cps1B. The corresponding construct $Cps1B_{32-1246}$-His_6 (144kDa) was cloned and purified as well as the N- and C-terminally truncated construct $Cps1B_{32-858}$-His_6 (99 kDa) lacking the TPR domain. M, marker; His_6, hexa-histidine tag.
