# Supplementary material for: A New Family of Capsule Polymerases Generates Teichoic Acid-Like Capsule Polymers in Gram-Negative Pathogens
Source: mBio. 2018 May 29;9(3):e00641-18. doi: 10.1128/mBio.00641-18 (PMC5974469; doi:10.1128/mBio.00641-18)
Supplement: FIG S8 [file mbo003183904sf8.pdf]

Fig. S8

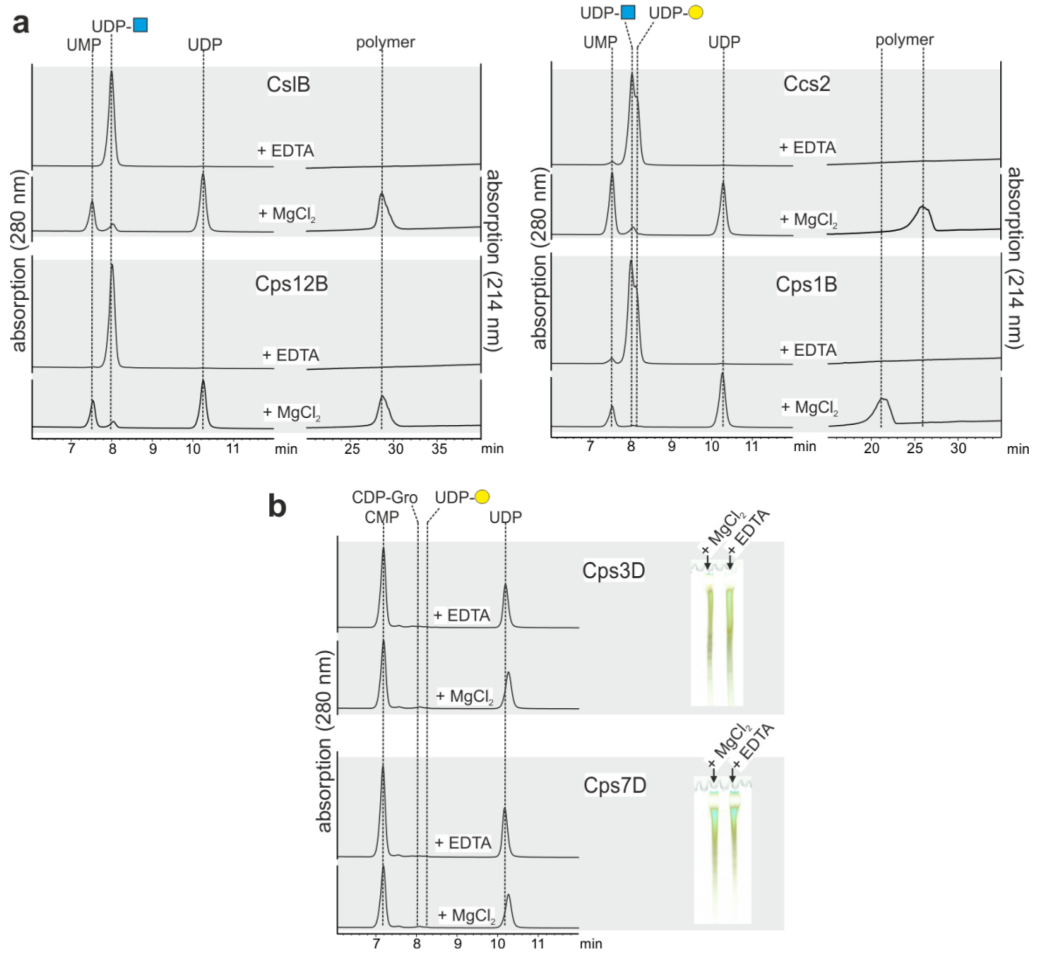

Fig. S8: HPLC-AEC assay in the presence (+$MgCl_2$) and absence (+EDTA) of magnesium chloride. a Polymerases containing GT-A-folded domains depend on Mg^{2+}, most likely to stabilize the negative charge of the diphosphate of their donor substrate (C. Breton, L. Snajdrová, C. Jeanneau, J. Koca, A. Imberty, *Glycobiology 16*:29R–37R, 2006). **b** Consistent with the fact that there is no evidence of a bound metal ion associated with catalysis in GT-B folded enzymes (C. Breton, L. Snajdrová, C. Jeanneau, J. Koca, A. Imberty, *Glycobiology 16*:29R–37R, 2006), polymerases adopting the TagF-like/GT-B architecture also work in the presence of the chelating agent EDTA.
