# Supplementary material for: A New Family of Capsule Polymerases Generates Teichoic Acid-Like Capsule Polymers in Gram-Negative Pathogens
Source: mBio. 2018 May 29;9(3):e00641-18. doi: 10.1128/mBio.00641-18 (PMC5974469; doi:10.1128/mBio.00641-18)
Supplement: FIG S9 [file mbo003183904sf9.pdf]

Fig. S9

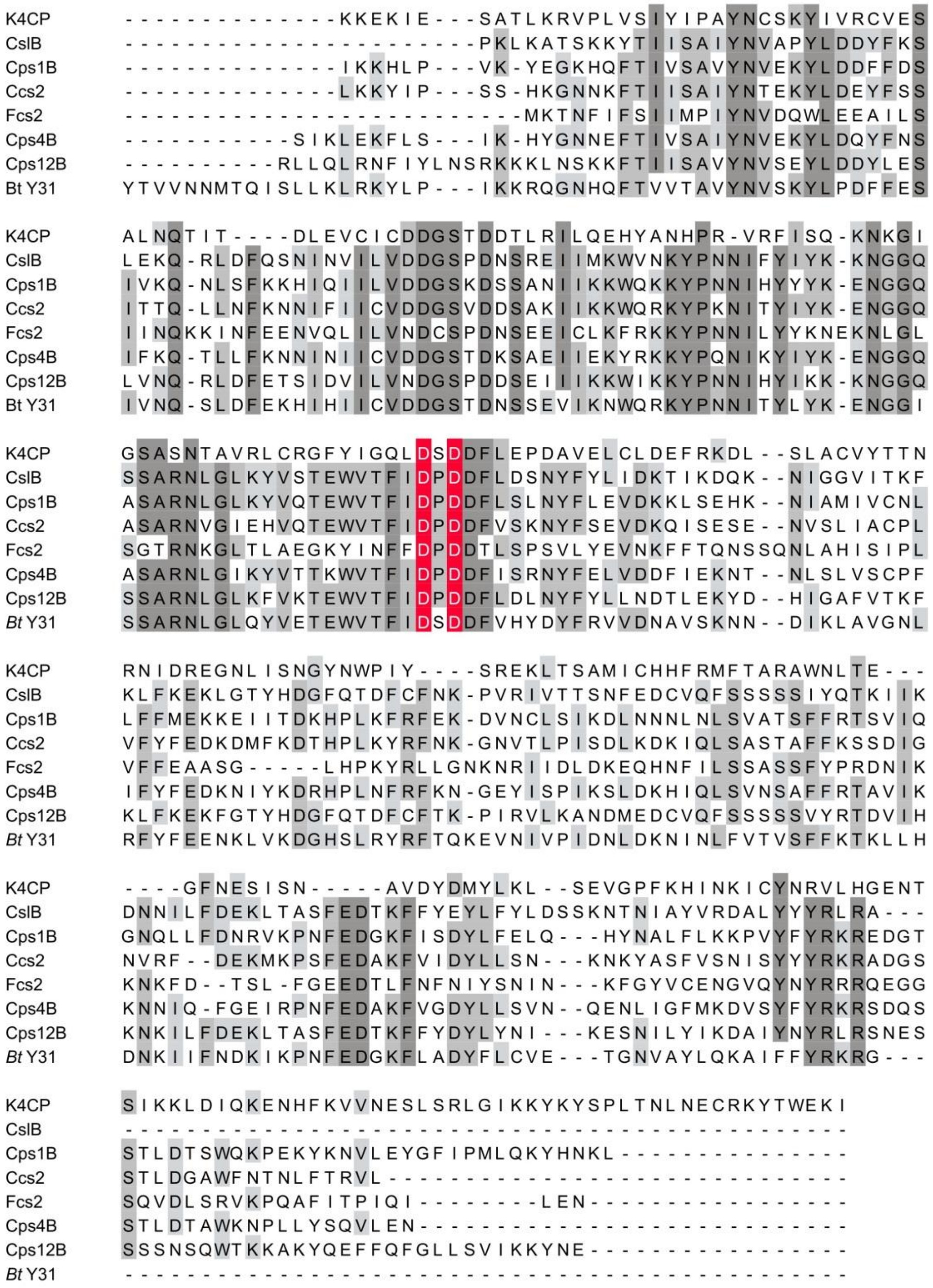

Fig. S9: Sequence alignment of all predicted N-terminal GT-A domains analyzed in this study including the sequence of the template K4CP (uniprot: Q8L0V4) used for PHYRE2 modeling. Database references for all TagF-like polmyerase sequences are indicated in the legend of Fig. S3. Identical amino acids are shown in grey boxes and aspartate residues of the conserved DxD motif are highlighted in red. The sequence alignment was performed with Clustal Omega (F. Sievers, A. Wilm, D. Dineen, T. J. Gibson, K. Karplus, W. Li, R. Lopez, H. McWilliam, M. Remmert, J. Söding, J. D. Thompson, D. G. Higgins, Mol Syst Biol 7:539, 2011) on the uniprot website (http://www.uniprot.org/align/) (E. Boutet, D. Lieberherr, M. Tognolli, M. Schneider, A. Bairoch, Methods Mol Biol 406:89–112, 2007) and annotated with the Jalview software (A. M. Waterhouse, J. B. Procter, D. M. A. Martin, M. Clamp, G. J. Barton, Bioinformatics 25:1189–91, 2009).
